# Genome-Wide Survey and Developmental Expression Mapping of Zebrafish SET Domain-Containing Genes

**DOI:** 10.1371/journal.pone.0001499

**Published:** 2008-01-30

**Authors:** Xiao-Jian Sun, Peng-Fei Xu, Ting Zhou, Ming Hu, Chun-Tang Fu, Yong Zhang, Yi Jin, Yi Chen, Sai-Juan Chen, Qiu-Hua Huang, Ting Xi Liu, Zhu Chen

**Affiliations:** 1 State Key Laboratory of Medical Genomics, Shanghai Institute of Hematology, Ruijin Hospital, Shanghai Jiao Tong University School of Medicine, Shanghai, China; 2 Institute of Health Sciences, Shanghai Institutes for Biological Sciences, Chinese Academy of Sciences and Shanghai Jiao Tong University School of Medicine, Shanghai, China; 3 Shanghai Center for Systems Biomedicine, Shanghai Jiao Tong University, Shanghai, China; 4 Model Organism Division, E-Institutes of Shanghai Universities, Shanghai, China; Deutsches Krebsforschungszentrum, Germany

## Abstract

SET domain-containing proteins represent an evolutionarily conserved family of epigenetic regulators, which are responsible for most histone lysine methylation. Since some of these genes have been revealed to be essential for embryonic development, we propose that the zebrafish, a vertebrate model organism possessing many advantages for developmental studies, can be utilized to study the biological functions of these genes and the related epigenetic mechanisms during early development. To this end, we have performed a genome-wide survey of zebrafish SET domain genes. 58 genes total have been identified. Although gene duplication events give rise to several lineage-specific paralogs, clear reciprocal orthologous relationship reveals high conservation between zebrafish and human SET domain genes. These data were further subject to an evolutionary analysis ranging from yeast to human, leading to the identification of putative clusters of orthologous groups (COGs) of this gene family. By means of whole-mount mRNA *in situ* hybridization strategy, we have also carried out a developmental expression mapping of these genes. A group of maternal SET domain genes, which are implicated in the programming of histone modification states in early development, have been identified and predicted to be responsible for all known sites of SET domain-mediated histone methylation. Furthermore, some genes show specific expression patterns in certain tissues at certain stages, suggesting the involvement of epigenetic mechanisms in the development of these systems. These results provide a global view of zebrafish SET domain histone methyltransferases in evolutionary and developmental dimensions and pave the way for using zebrafish to systematically study the roles of these genes during development.

## Introduction

Nucleosome, consisting of DNA wrapped around an octamer of histone proteins, not only acts as an elementary unit of eukaryotic chromatin packaging but also plays an active role in regulation of gene expression and other aspects of chromatin functions [1]. Covalent modifications of histones (acetylation, methylation, phosphorylation, ubiquitination, etc.) have emerged as key regulatory mechanisms of transcriptional regulation and may serve as an epigenetic marking system that is responsible for establishing and maintaining the heritable programs of gene expression during cellular differentiation and organism development [2]–[4]. Recently, a “histone code” hypothesis has been proposed to explain how different histone modifications can result in distinct chromatin-regulated functions [5], [6]. Various enzymes that are responsible for labeling and erasing the histone modifications (“writers”) and proteins that specifically recognize these modifications (“readers”) play a key role in the process of translating the “histone code” [4]. Histone modifications have been thought to be highly conserved through evolution, based on several supporting facts: 1) the core histones, originating before the divergence of the archaeal and eukaryotic lineages, exist in all eukaryotic organisms [7], [8]; 2) the amino acid sequences and modification sites of the histones are highly conserved [9]; and 3) families of specific enzymes that modify the histones are widespread in eukaryotic genomes [10], [11]. However, a recently reported examination of the universalness of “histone code” reveals significant differences of histone modification patterns among species, and meanwhile, several potentially species-specific histone modifications and several novel histone modifications have been observed [12]. These differences are at least partially due to the evolutionary diversities of histone-modifying enzymes. Therefore, an extensive evolutionary analysis of these enzymes should contribute to deciphering the further complicated “histone code”.

A family of SET domain-containing proteins catalyzes methylations of histone lysine residues, with only exception of H3 lysine 79 [13], [14]. The SET domain was originally identified in members of polycomb group (PcG), trithorax group (trxG), and Su(var) genes and was named after the genes *Su(var)3-9*, *Enhancer of zeste* (*E(z)*) and *trithorax* (*trx*) [15]. Much has been learned regarding the biochemical characterization of the histone methyltransferase (HMT) activities of the SET domain proteins and their effects on both gene repression and gene activation [13], [14]. However, the functions of these HMTs during development are still largely unclear [16]. In the early development of vertebrates from the stages of cleavage through blastulation and gastrulation to organogenesis, gene expression is subject to a high degree of temporal and spatial regulation, and the levels and locations of histone modifications are also dynamically changed [17], [18]. Accordingly, recent genetic studies indicate that some SET domain genes are essential for normal embryo development and survival [19]–[25]. Therefore, we propose that the zebrafish, an ideal model organism for studying vertebrate development [26], [27], can be utilized to study the biological functions of these genes during early development. The particular advantages of zebrafish, such as the high fecundity, rapid external development and the extraordinary optical clarity of the embryos, allow easy analysis of histone modifications and gene expressions by means of immunostaining and whole-mount *in situ* hybridization (WISH) strategies. Particularly, our immunofluorescent analyses of zebrafish embryos with histone modification-specific antibodies reveal that histone H3 lysine 36 (H3K36) methylation firstly emerges at 64-cell stage, immediately after the phosphorylation of RNA polymerase II (pol II) (Figure S1), consistent with the previously described physical association between an H3K36-specific HMT HYPB/SETD2 and the hyperphosphorylated pol II [28]. These observations suggest that zebrafish embryos can be used as a tool to study the mechanism of histone modification in the context of development, and demonstrate the strength of a wide-scale expression survey to identify the master epigenetic regulator genes. Furthermore, given that a number of SET domain genes are implicated in human diseases, notably cancers [29], [30], a zebrafish model that mimics the mechanisms of human cancer would be invaluable for large-scale screens for cancer modifiers, and simultaneously, for targeted-therapeutic drugs [31].

To gain an overall insight into zebrafish SET domain genes and to evaluate the evolutionary conservation of them with their human counterparts, we firstly performed a genome-wide survey of SET domain genes of zebrafish, followed by an evolutionary analysis of these genes between zebrafish and human. Considering zebrafish as a representative organism of a lower vertebrate [32], these results not only provide an outline of evolutionary history of this gene family in vertebrate, but also allow gaining a more extensive view of the conservation and diversity of these genes in organisms ranging from yeast to human, which would contribute to the explanation of the recently discovered organismal differences in histone modifications [12]. Meanwhile, we performed WISH analyses to obtain a developmental expression profile of the zebrafish SET domain genes. The WISH assay with zebrafish makes it easy to detect the expression pattern of a gene during embryonic development, therefore it has been widely used to explore gene function [33], [34]. Furthermore, merging the information of evolutionary histories, structural features and developmental expression patterns of these genes should provide insights into their biological functions and underlying mechanisms. Although the dynamics of histone modifications in zebrafish early development has not been well described, the conservation of SET domain HMT genes between zebrafish and human, in both structures and expression patterns, suggests that the mechanisms of the SET domain-mediated histone lysine methylations are highly conserved between the two species. Taken together, these analyses support zebrafish as an ideal model organism for systematically studying the roles of the SET domain genes during development.

## Results

### Identification of zebrafish SET domain genes

Considering that the known zebrafish SET domain genes and the zebrafish EST data are not yet sufficient for a genome-wide analysis, we utilized the zebrafish whole-genome shotgun trace data to survey the SET domain genes. The human and fruit fly (*Drosophila melanogaster*) SET domains were used as queries because these two organisms are representative of vertebrate and invertebrate, respectively, and possess relatively complete genomic information. By searching the SMART (Simple Modular Architecture Research Tool) domain database [35] and PSI-BLAST [36] analysis of NCBI (National Center of Biotechnology Information) non-redundant protein database, followed by extracting the chromosomal location of the entries and removing the redundancies, 47 and 29 SET domain proteins of human and fruit fly were obtained (Table S1). Use of alternative databases such as Pfam [37] and PROSITE [38] provided consistent results. To identify the SET domain genes from zebrafish genome, the sequences of the SET domains in both human and fruit fly were used for independent TBLASTN searches against the zebrafish whole-genome shotgun trace database. The retrieved entries were clustered and aligned with CAT (Clustering and Alignment Tools) program [39], successively followed by manual edit, alignment with zebrafish genome assemblies, gene prediction with GENSCAN program [40], and BLAST search of zebrafish EST database. As a result, totally 58 non-redundant zebrafish SET domain genes were identified, and their putative chromosomal locations were mapped according to the latest zebrafish genome mapping information (Table 1). Unexpectedly, the result clearly demonstrates that zebrafish carries more SET domain genes than human (see below for more analyses).

**Table 1:** Zebrafish SET domain genes

Gene Name	Description	GenBank Accession Number	Subfamily	Chromosome Number	Closest Human Homolog
*ehmt2*	euchromatic histone lysine N-methyltransferase 2	DQ840136	I	19	*EHMT2*
*ehmt1b*	euchromatic histone methyltransferase 1b	DQ355788*, DQ840137	I	21	*EHMT1*
*ehmt1a*	euchromatic histone methyltransferase 1a	DQ840138	I	5	*EHMT1*
*suv39h1b*	suppressor of variegation 3-9 homolog 1b (Drosophila)	DQ840139	I	8	*SUV39H1*
*suv39h1a*	suppressor of variegation 3-9 homolog 1a (Drosophila)	DQ840140	I	8	*SUV39H1*
*setdb2*	SET domain, bifurcated 2	DQ358104*	I	1	*SETDB2*
*setdb1b*	SET domain, bifurcated 1b	DQ358103*, DQ840141	I	16	*SETDB1*
*setdb1a*	SET domain, bifurcated 1a	DQ840142	I	19	*SETDB1*
*setmar*	SET domain and mariner transposase fusion gene	DQ840143	II	11	*SETMAR*
*ash1l*	ash1 (absent, small, or homeotic)-like (Drosophila)	DQ840144	III	19	*ASH1L*
*setd2*	SET domain containing 2	DQ343298*, DQ840145	III	16	*SETD2*
*nsd1b*	nuclear receptor binding SET domain protein 1b	DQ840146	III	21	*NSD1*
*nsd1a*	nuclear receptor binding SET domain protein 1a	DQ840147	III	14	*NSD1*
*whsc1*	Wolf-Hirschhorn syndrome candidate 1	DQ358102*, DQ840148	III	13	*WHSC1*
*whsc1l1*	Wolf-Hirschhorn syndrome candidate 1-like 1	DQ840149	III	10	*WHSC1L1*
*ezh2*	enhancer of zeste homolog 2 (Drosophila)	DQ840150	IV	24	*EZH2*
*ezh1*	enhancer of zeste homolog 1 (Drosophila)	DQ840151	IV	3	*EZH1*
*mll2*	myeloid/lymphoid or mixed-lineage leukemia 2	DQ840152	V	23	*MLL2*
*mll3a*	myeloid/lymphoid or mixed-lineage leukemia 3a	DQ840153	V	24	*MLL3*
*mll3b*	myeloid/lymphoid or mixed-lineage leukemia 3b	DQ840154	V	2	*MLL3*
*mll4b*	myeloid/lymphoid or mixed-lineage leukemia 4b	DQ840155	V	15	*MLL4*
*mll4a*	myeloid/lymphoid or mixed-lineage leukemia 4a	DQ840156	V	19	*MLL4*
*mll*	myeloid/lymphoid or mixed-lineage leukemia (trithorax homolog, Drosophila)	DQ355790*, DQ355791*, DQ840157	V	15	*MLL*
*setd1a*	SET domain containing 1A	DQ355789*, DQ851808	V	3	*SETD1A*
*setd1ba*	SET domain containing 1Ba	DQ851809	V	10	*SETD1B*
*setd1bb*	SET domain containing 1Bb	DQ851810	V	11	*SETD1B*
*setd8b*	SET domain containing 8b	DQ851825	VI	5	*SETD8*
*setd8a*	SET domain containing 8a	DQ343297*, DQ851826	VI	10	*SETD8*
*setd7*	SET domain containing 7	DQ851811	VII	14	*SETD7*
*setd5*	SET domain containing 5	DQ851812	VIII	6	*SETD5*
*mll5*	myeloid/lymphoid or mixed-lineage leukemia 5 (trithorax homolog, Drosophila)	DQ851813	VIII	4	*MLL5*
*suv420h1*	suppressor of variegation 4-20 homolog 1 (Drosophila)	DQ851814	IX	18	*SUV420H1*
*suv420h2*	suppressor of variegation 4-20 homolog 2 (Drosophila)	DQ851815	IX	3	*SUV420H2*
*setd6*	SET domain containing 6	DQ851816	IX	25	*SETD6*
*smyd5*	SET and MYND domain containing 5	DQ851817	IX	14	*SMYD5*
*smyd4*	SET and MYND domain containing 4	DQ851818	IX	10	*SMYD4*
*smyd1b*	SET and MYND domain containing 1b	DQ851819	IX	8	*SMYD1*
*smyd1a*	SET and MYND domain containing 1a	DQ851820	IX	5	*SMYD1*
*smyd3*	SET and MYND domain containing 3	DQ851821	IX	17	*SMYD3*
*smyd2b*	SET and MYND domain containing 2b	DQ851822	IX	3	*SMYD2*
*smyd2a*	SET and MYND domain containing 2a	DQ851823	IX	17	*SMYD2*
*prdm16*	PR domain containing 16	DQ851827	X	8	*PRDM16*
*prdm3*	PR domain containing 3	DQ851828	X	15	*PRDM3*
*prdm5*	PR domain containing 5	DQ851829, EU258933	X	n.a.	*PRDM5*
*prdm2*	PR domain containing 2	DQ851830	X	11	*PRDM2*
*prdm8b*	PR domain containing 8b	DQ851833	X	21	*PRDM8*
*prdm8a*	PR domain containing 8a	DQ851834	X	13	*PRDM8*
*prdm13*	PR domain containing 13	DQ851835	X	16	*PRDM13*
*prdm9*	PR domain containing 9	DQ851831	X	21	*PRDM7, PRDM9*
*prdm11*	PR domain containing 11	DQ851832	X	25	*PRDM11*
*prdm12*	PR domain containing 12	DQ851836	X	24	*PRDM12*
*prdm6*	PR domain containing 6	DQ851837, EU258934	X	n.a.	*PRDM6*
*prdm14*	PR domain containing 14	DQ851838	X	24	*PRDM14*
*prdm1a*	PR domain containing 1a	DQ851839	X	16	*PRDM1*
*prdm1b*	PR domain containing 1b	DQ851840	X	19	*PRDM1*
*prdm1c*	PR domain containing 1c	DQ851841, EU258932	X	5	*PRDM1*
*prdm15*	PR domain containing 15	DQ851842	X	10	*PRDM15*
*prdm4*	PR domain containing 4	DQ851843	X	4	*PRDM4*

* Clones derived from the zebrafish kidney cDNA library (Ref. 34).

n.a., not available.

To confirm the existence and the expression of these predicted genes, we cloned these genes with two strategies: 1) At least 8 zebrafish SET genes were found in our large-scale sequence data of the zebrafish kidney cDNA library described previously [34]. 2) All the zebrafish SET domain genes were cloned in certain fragments with RT-PCR amplification from zebrafish embryos or adults, followed by completely sequencing. The resulting sequences of both types of clones were further analyzed to localize the open reading frames (ORFs) and deduced into peptides, and the annotated sequences were submitted to the GenBank (accession numbers: DQ343297, DQ343298, DQ355788-DQ355791, DQ358102-DQ358104, DQ840136-DQ840157, DQ851808-DQ851843, EU258932-EU258934) (Table 1). These results indicate that the 58 zebrafish SET domain genes indeed exist and are naturally expressed. During the survey of these genes, notably, several possible pseudogenes were also observed; they usually contain a SET domain-like genomic region, which can be recognized by the TBLASTN analysis, but lack a valid ORF (e.g. the SET domain-like regions are disrupted by several stop codons) (data not shown).

### Phylogenetic analysis and classification of zebrafish and human SET domain genes

**Figure 1:**
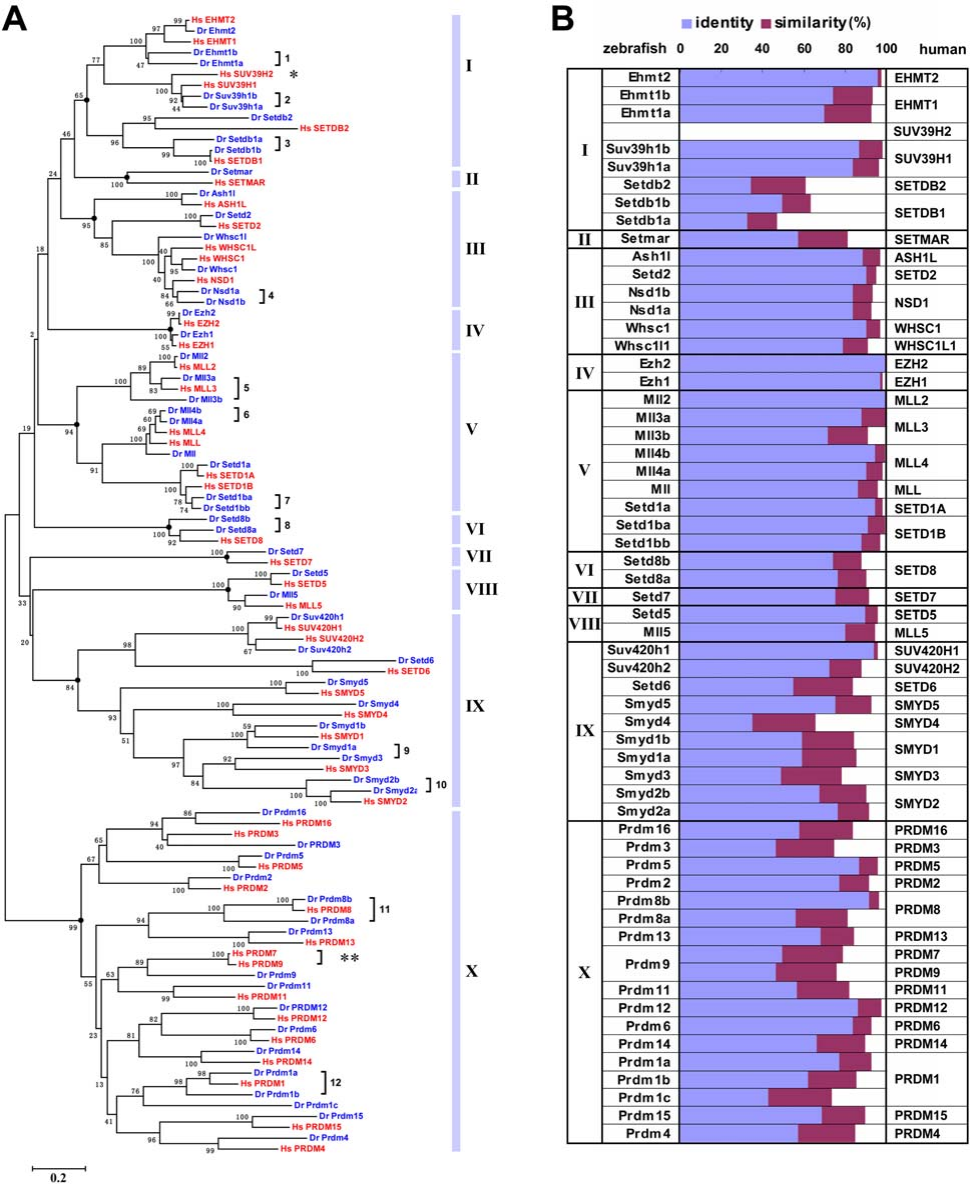
Evolutionary conservation of zebrafish and human SET domain genes. (A) Phylogenetic analysis. Unrooted neighbor-joining tree was constructed based on the alignment of the amino acid sequences of the SET domains of 47 human proteins (*red*) and 58 predicted zebrafish proteins (*blue*). Bootstrap percentages computed from 1000 replicates are shown along the internal braches. The major branches (bootstrap support ≥ 65%; labeled with black circles) define 10 subfamilies of the genes, which are denoted with light blue vertical bars. The single brackets followed by numbers denote zebrafish gene pairs that have been found corresponding to single human genes. Note that zebrafish likely lacks an ortholog of human *SUV39H2* gene (*single asterisk*) and that human *PRDM7* and *PRDM9* genes (*double asterisks*) are co-orthologous to a single zebrafish gene named *prdm9*. Abbreviations: Hs, *Homo sapiens*; Dr, *Denio rerio*. (B) One-to-one identities and similarities between the SET domains of zebrafish proteins and their human counterparts. The identities and similarities on SET domains were calculated with FASTA program (http://fasta.bioch.virginia.edu/fasta_www2) and represented with blue and purple bars, respectively.

The evolutionary relationships among the zebrafish and human SET domain genes were examined by phylogenetic analysis. As shown by the neighbor-joining tree that was constructed based on the alignment of the amino acid sequences of the SET domains of the encoded proteins [41] (Figure 1A), it is generally observed that a zebrafish SET domain gene and a human SET domain gene form a monophyletic branch (occasionally, two zebrafish genes are clustered together with a single human gene and thereby act as potential “zebrafish lineage-specific paralogs”, which will be elucidated below), suggesting reciprocal orthologous relationships between them. Considering zebrafish as a lower vertebrate organism, this phylogenetic analysis indicates a good conservation of SET domain genes through vertebrate evolution. Furthermore, according to this tree, the vertebrate SET domains are divided into 10 subfamilies (≥65% bootstrap support; if the cut-off bootstrap value is set higher than 80%, the subfamily I can be further divided into 3 groups). When using different methods (e.g. Minimum Evolution and Maximum Parsimony methods) to construct the trees [41], similar results were consistently reproduced.

In addition to the SET domains, it is worth while to note that most of these SET domain genes also carry a series of other functional domains, which are expected to direct the SET domain proteins to certain complexes and to mediate some specific activities. We therefore analyzed the domain architectures of the full-length human SET domain proteins and the predicted zebrafish SET domain proteins. As a result, the domain-architecture information is well consistent with the SET domain-based phylogeny, and both support the 10-subfamily definition (Figure S2). For example, MLL5 (myeloid/lymphoid or mixed-lineage leukemia 5) protein has been classified into subfamily VIII because its SET domain shows high homology with SETD5 rather than its “paralogs” in subfamily V (i.e. MLL, MLL2, MLL3 and MLL4) (Figure 1A). Consistent with this result, the domain architecture analysis demonstrated that MLL5 protein lacks a PostSET domain that is a common characteristic of the members of subfamily V (Figure S2), thus supporting the SET domain-based classification. Furthermore, this correlativity between SET domains and the domain architectures suggests that the other domains and their arrangement significantly contribute to the evolution of SET domain *per se*. Therefore, characterization of functional domains (e.g. DNA binding domains and protein-protein interaction domains) and the domain architectures is important for understanding the function of these SET domain genes. In particular, several members of subfamilies IX and X (e.g. PRDM1 [42], PRDM2 [43] and SMYD3 [44]) have been proven to be transcription factors that directly bind to certain DNA elements, whereas some other SET domain proteins (e.g. SUV39H1 [45], G9a/EHMT1 [46], SETDB1 [47] and EZH2 [48]) are usually recruited by certain transcription factors, and thereby function as cofactors in transcriptional machineries. These observations imply that the SET domain proteins may function in at least two manners (transcription factors versus cofactors).

To further examine the conservation between zebrafish and human SET domain genes, we employed FASTA program [49] to perform a one-to-one comparison of their SET domains. As shown in Figure 1B, the zebrafish genes show rather high identities/similarities with their human counterparts, further supporting the conservation of vertebrate SET domain genes. SETDB1 and SETDB2 in subfamily I show relatively low identities/similarities, largely due to the SET domains of these proteins are disrupted by an inserted sequence that is not well conserved between the two species [47]. Since all the zebrafish genes and their human counterparts emerged simultaneously at the divergence of teleost and the ancestor of mammals approximately 450 million years ago [32], the different identities/similarities reflect the different selective pressure for the biological processes these genes involved in. Notably, members of subfamilies III, IV and V show relatively high identities/similarities, whereas those of subfamilies IX and X show moderate ones, implying that these different subfamilies of genes may function distinctly in each species.

### Origins of zebrafish lineage-specific SET domain genes

**Figure 2:**
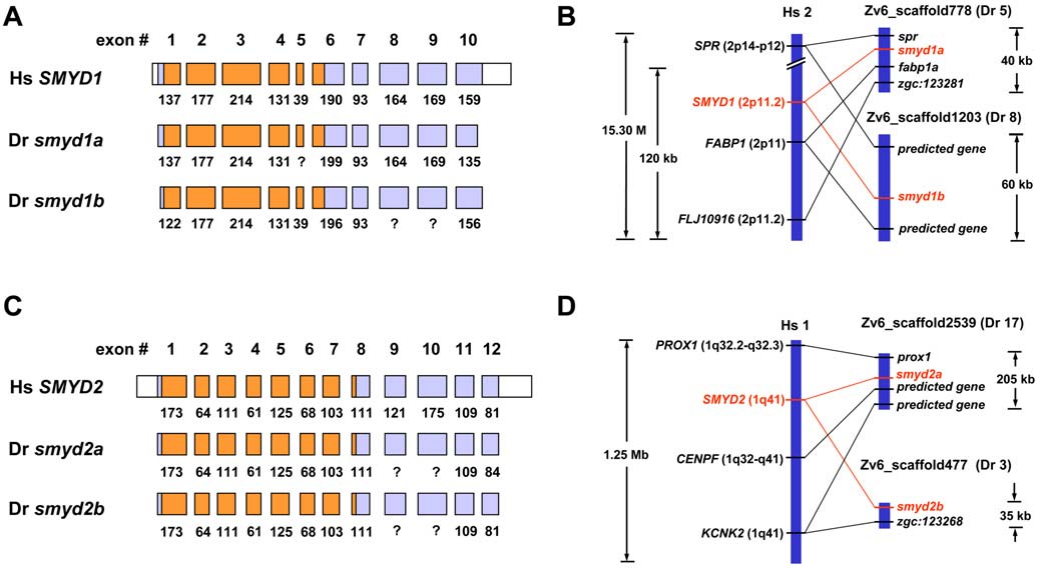
Genomic analysis of two pairs of zebrafish SET domain genes and their human counterparts. (A and C) Exon-intron structures of human *SMYD1* and *SMYD2* genes and zebrafish *smyd1a*, *smyd1b*, *smyd2a* and *smyd2b* genes. The exons are drawn to scale and the numbers beneath them indicate the size in bases. The question marks indicate several exons whose size can not be determined precisely. (B and D) Comparison of the SET domain gene loci in zebrafish and human genome reveals conserved syntenies. See Figure S3 for more analyses. The SET domain genes are indicated in *red* while the neighboring genes in *black*. Chromosome numbers of human (*Hs*) and zebrafish (*Dr*) are shown. The chromosomal locations of human genes are shown in parentheses after the gene names. Distances between genes on a single chromosome are shown to scale, and the compared chromosomes are scaled to equivalent lengths. Lines between the compared chromosomes connect positions of orthologous gene pairs in the two species. Several zebrafish genes were predicted according to GENSCAN analysis of zebrafish genome contigs and EST alignments.

When analyzing the homology between zebrafish and human SET domain genes, we frequently found that a pair of zebrafish genes showed high homology to a single human gene. These gene pairs were thereby named with *a* and *b* after the gene symbols (e.g. *suv39h1a* and *suv39h1b*; Table 1 and Figure 1). In this study, totally 12 zebrafish-specific gene pairs have been identified (Figure 1A, indicated with single brackets and numbers), which largely leads to the fact that zebrafish carries more SET domain genes than human. Generally, the zebrafish-specific gene pairs may result from zebrafish lineage-specific gene duplication or human lineage-specific gene loss, or both. In view of the facts that 1) we did not find the same gene pairs in other tetrapod (e.g. mouse, rat or frog, etc.), and 2) a whole-genome duplication (WGD) and a subsequent massive loss of duplicated genes occurred in the teleost has been suggested by several lines of evidences [50]–[52], we hypothesize that these zebrafish-specific gene pairs were raised through the teleost lineage-specific WGD and therefore collectively orthologous (“co-orthologous”) to their human counterparts. To address this issue, we firstly analyzed the genomic structures of these genes in terms of exon/intron organization patterns in combination with the domain architectures. Most of the exons of these zebrafish genes can be identified from the genomic contigs, although the size of some exons can not be determined precisely, largely due to the gaps in the zebrafish genomic contigs or relatively low homologies of these exons between zebrafish and human. These exon/intron structural analyses provided useful information for determining the evolutionary relationships among these genes. For example, zebrafish *smyd1a* (GenBank accession DQ851820) and *smyd1b* (GenBank accession DQ851819) genes show exactly identical exon/intron structures with human *SMYD1* gene: 10 exons with a SET domain located in the exons 1-6 (Figure 2A). In contrast, the *SMYD2*-group genes, including zebrafish *smyd2a* (GenBank accession DQ851823), *smyd2b* (GenBank accession DQ851822) genes and human *SMYD2* gene, have 12 exons with a SET domain located in the exons 1-8 (Figure 2C). On the other hand, we employed syntenic analysis to further determine the orthologous relationship of these genes. As a result, for example, zebrafish *smyd1a* and *smyd1b* genes are located on two distinct zebrafish genomic contigs Zv6_scaffold778 and Zv6_scaffold1203, which are assigned to different chromosomes according to the current version of genome assembly (Figure 2B and D). Note that several of their close neighboring genes also have putative human orthologs located near the human *SMYD1* gene on the long arm of chromosome 2, indicating highly conserved syntenies between the two species. Among these genes, interestingly, more than one zebrafish gene pairs were observed to be corresponding to single human genes (Figure 2B and D), suggesting that these syntenies are generated by genome-scale duplication instead of random gene duplication. In addition, we extensively analyzed all 12 zebrafish gene pairs and the conserved syntenies between zebrafish and human were observed for most genes, although the exceptional cases of *mll4a* (GenBank accession DQ840156) and *prdm1b* (GenBank accession DQ851840) genes require further analysis to reconstruct their evolutionary history (Figure 2B and D and Figure S3). Taken together, independent evidences (i.e. phylogenetic relationship, identical exon/intron structures and conserved syntenies) strongly support that the zebrafish-specific gene pairs were raised from a genome-scale duplication event and therefore co-orthologous to their human counterparts.

### Origins of human lineage-specific SET domain genes

**Figure 3:**
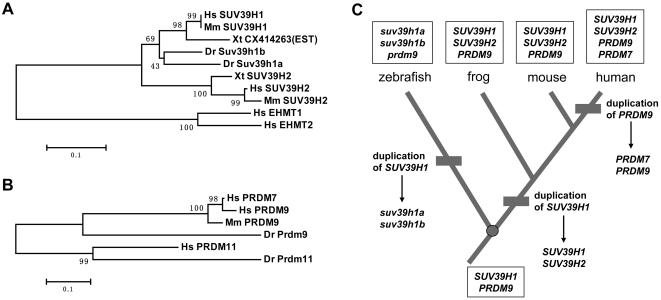
Evolution of human lineage-specific SET domain genes. (A) Phylogenetic tree of human SUV39H1, SUV39H2 proteins and their closest homologs in mouse (*Mus musculus*, *Mm*), frog (*Xenopus tropicalis*, *Xt*) and zebrafish is constructed based on alignment of the amino acid sequences of their SET domains. Human EHMT1 and EHMT2 proteins were used as an outgroup to root the tree. Bootstrap percentages computed from 1000 replicates are shown along the internal braches. (B) Phylogenetic tree of human PRDM7, PRDM9 proteins and their closest homologs in mouse and zebrafish. Human PRDM11 and zebrafish Prdm11 proteins were used as an outgroup to root the tree. (C) Schematic representation of the possible evolutionary history of vertebrate *SUV39H1*, *SUV39H2*, *PRDM7* and *PRDM9* genes. Three gene duplication events through evolution, which give rise to gene pairs in certain lineages, are shown as short bars along the branches, and the common ancestor is depicted as a filled circle. Genes of certain species and the common ancestor are written in boxes.

The phylogenetic analysis of zebrafish and human SET domain genes reveals 2 pairs of potential human lineage-specific paralogs. 1) While human *SUV29H1* gene definitely has a pair of co-orthologs in zebrafish (i.e. *suv39h1a* (GenBank accession DQ840140) and *suv39h1b* (GenBank accession DQ840139)) as described above, its closest paralog, the human *SUV39H2* gene, appears to lack a zebrafish ortholog (Figure 1A; asterisk). 2) A pair of human genes *PRDM7* and *PRDM9*, located on chromosomal regions 16q24.3 and 5p14, respectively, are corresponding to a single zebrafish gene herein named *prdm9* (GenBank accession DQ851831) (Figure 1A; double asterisks). To figure out the origin of these human genes, extensive database searches were performed and the resulting sequences were subject to phylogenetic analyses. Interestingly, the results indicate different evolutionary histories of these 2 pairs of human genes. The *SUV39H2* gene is found in tetrapod (e.g. human, mouse and frog) but not in zebrafish (Figure 3A), suggesting that this gene is likely generated by a tetrapod lineage-specific duplication event. In contrast, although human possesses a *PRDM7* gene and a *PRDM9* gene, other vertebrates ranging from zebrafish to mouse just have a single gene, named *PRDM9* herein (Figure 3B), suggesting that this pair of human-specific paralogs are result from a gene duplication event after the divergence of the ancestors of human and mouse. Taken together, these data suggest that two different duplication events gave rise to the human lineage-specific paralogs *SUV39H1*/*SUV39H2* genes and *PRDM7*/*PRDM9* genes (Figure 3C).

### Clusters of Orthologous Groups (COGs) of SET domain genes ranging from yeast to human

**Figure 4:**
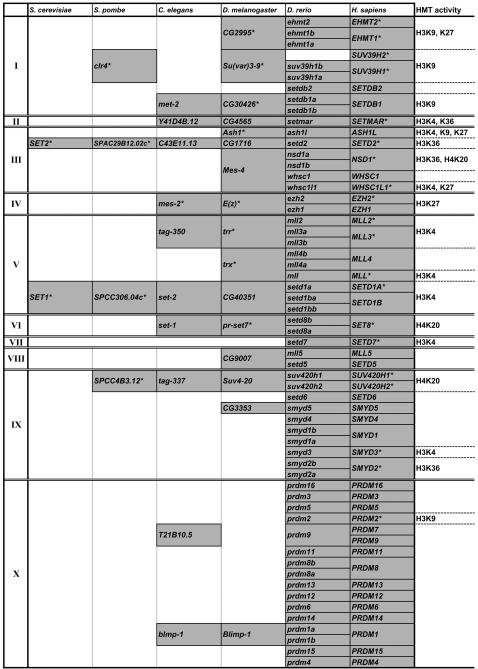
Clusters of orthologous groups (COGs) of SET domain genes from yeast to human. The relationship was determined based on combined information of “reciprocal best hit” analysis, phylogenetic analysis, and synthenic analysis. Note that, occasionally, two or more genes in one species are collectively orthologous to one gene in another species. These genes are defined as co-orthologs and incorporated into a same COG. Known histone methyltransferases (HMTases) are denoted with asterisks and their site specificities are indicated along the corresponding COGs.

Besides those in vertebrates, a number of SET domain genes from invertebrate animals and fungi have been identified and functionally characterized (see Table S1 for a summary of the so far characterized SET domain proteins with specific HMTase activities). Cross-species comparison of these genes would be helpful to build a comparative framework and to bridge barriers among organism-based research communities. Particularly, determination of evolutionary relationship and identification of clusters of orthologous groups (COGs) is useful to delineate functions of the corresponding genes in different species [53]. To this end, we extracted a number of SET domain genes from human (47), *Drosophila* (29), *C. elegans* (30), *S. pombe* (11) and *S. cerevisiae* (7) through analyses of SMART database and NCBI protein database (Table S1). Among these genes, COGs were identified based on multiple approaches: 1) “reciprocal best hits” algorithm, a straightforward method for prediction of one-to-one orthologs [34]. However, lineage-specific gene duplications (and also asymmetrical evolution of paralogs sometimes) likely lead to false negatives under this method [53]. 2) Phylogenetic analysis (Figure S4) in combination with tree reconciliation, which is useful to complement the limitation of the “reciprocal best hits” method. Under this approach, the orthologous relationship is reflected by the comparison and reconciliation between the topology of a gene tree and that of the chosen species tree [53]. 3) Genomic structure comparison that relies on the assumption that the ancestral structure (exon/intron patterns) and order (syntenies) of orthologous genes are retained in the genomes of descendent species [54]. As a result, a set of COGs of SET domain genes were identified (Figure 4), which has a special reference to the functional characteristics of these genes, and may also contribute to outlining an evolutionary history of them. For example, we hereby tried to apply this result to address a question about the origins of the site specificities of SET domain HMTs through evolution (see discussion).

### Developmental expression mapping of zebrafish SET domain genes

While the structural and syntenic comparisons, phylogenetic analyses and COG identifications presented above outline the histories of the SET domain genes and contribute to understanding their functions in the context of evolution, developmental expression analysis can reveal more properties of zebrafish SET domain genes in the context of development, in which epigenetic mechanisms have been suggested to play an important role. During the early development of zebrafish embryos, several major developmental and cellular processes (including initiation of zygotic transcription, differentiation of three germ layers and organogenesis) occur by 72 hour postfertilization (hpf) [55]. Therefore, we chose zebrafish embryos at 0.75, 2, 4, 6, 9, 18, 24, 48, 72 and 120 hpf for WISH analysis to determine the expression of SET domain genes. The sequences corresponding to all probes used in this study were deposited into the GenBank (Table 1).

Zygotic transcription of zebrafish initiates at approximately cell cycle 10-13 (3–4 hpf) that termed midblastula transition (MBT), and before which, all developmental processes (e.g. fertilization, egg activation, early cell division and the initiation of zygote transcription) must rely on maternally deposited gene products [56], [57]. To gain clues to the roles of SET domain genes in these early developmental processes, we compared the expression levels of them in embryos at 0.75, 2 and 4 hpf with WISH analyses (Figure S6) and identified the highly expressed SET domain genes (Table 2). After the MBT, the expression levels of most maternally deposited SET domain gene transcripts significantly decreased (data not shown), probably due to mRNA turnover [58]. Thus, the staining signals in embryos at later stages mostly reflect the expression of genes in zygote genome. Among them, 13 out of 58 SET domain genes (22.4%) were observed to have specific expression patterns in at least on stage, whereas other SET domain genes were found ubiquitously expressed. Notably, among the ubiquitously expressed genes, a number of them show relatively higher expression in certain tissues (e.g. central nervous system, intermediate cell mass of mesoderm, etc.; Figure S5). From previous literatures, we can find several mammalian SET domain genes that have been determined with expression analyses (e.g. Northern blot or RT-PCR assays), and those data are largely consistent with our WISH analyses of zebrafish SET domain genes (see below for examples). Furthermore, we compared our data with the mRNA *in situ* hybridization analyses of some mouse SET domain genes deposited in the Mouse Genome Informatics database (http://www.informatics.jax.org/). Although some high resolution section analyses with mouse tissues are not sufficient for providing global views, high similarities in the expressions of the orthologous genes in zebrafish and mouse were observed (Table S2). Taken together, these data suggest the conservation of the expressions of the vertebrate SET domain genes.

**Table 2:** Maternally expressed SET domain genes

Gene	Subfamily	Orthologs			HMT activity
		fruit fly	worm	yeast	
*ehmt1b*	I	+			H3K9,K27
*suv391a*	I	+		+	H3K9
*setdb1a*	I	+	+		H3K9
*setd2*	III	+	+	+	H3K36
*whsc1*	III	+			?
*ezh2*	IV	+	+		H3K27
*mll4a*	V	+			?
*mll*	V	+			H3K4
*setd1a*	V	+	+	+	H3K4
*setd8b*	VI	+	+		H4K20
*suv420h1*	IX	+	+	+	H4K20
*suv420h2*	IX	+	+	+	H4K20
*prdm2*	X				H3K9
*prdm9*	X		+		?
*prdm14*	X				?

“+” denotes an ortholog of the indicated gene was found in the species (i.e. fruit fly, worm or yeast).

### Maternally expressed SET domain genes

Fifteen maternally expressed SET domain genes were identified by WISH analyses (Figure S6 and Table 2). These genes show high expression levels in the embryos at early stages, especially at 0.75 and 2 hpf (Figure S6). By merging information on their classifications, evolutionary histories and HMT specificities, several properties of this group of maternal SET domain genes were observed (Table2): 1) These genes distribute in 7 subfamilies (I, III, IV, V, VI, IX and X) while no significant subfamily-discrimination was observed. 2) They are relatively conserved in that 13 out of 15 genes have at least one ortholog in fruit fly, worm or yeast. 3) They are predicted to be responsible for all the known SET domain-mediated histone methylations (i.e. H3K4me, K9me, K27me, K36me and H4K20me). These observations can be applied to understanding the potential role of these genes in the programming of histone modifications during early embryogenesis. For example, immunofluorescent staining of zebrafish embryos revealed that histone H3K36 methylation firstly emerges at approximately 64-cell (2 hpf) stage (Figure S1). In view that only one potential H3K36 HMT gene, *setd2* (GenBank accession DQ343298 and DQ840145), was significantly expressed from 0.75 to 2 hpf (Table 2), we hypothesize that Setd2 HMT may catalyze the H3K36 methylation during the early development. Furthermore, in mouse embryos, dynamic changes of histone lysine methylation have been described to characterize the first cell cycle, which takes place prior to the zygotic transcription [59]. Given the conservation of SET domain HMTs in vertebrates, zebrafish embryos may also carry these kinds of epigenetic changes during early development, in which the maternally deposited transcripts of SET domain genes are likely to play an important role.

### Somite/muslce-expressed SET domain genes

**Figure 5:**
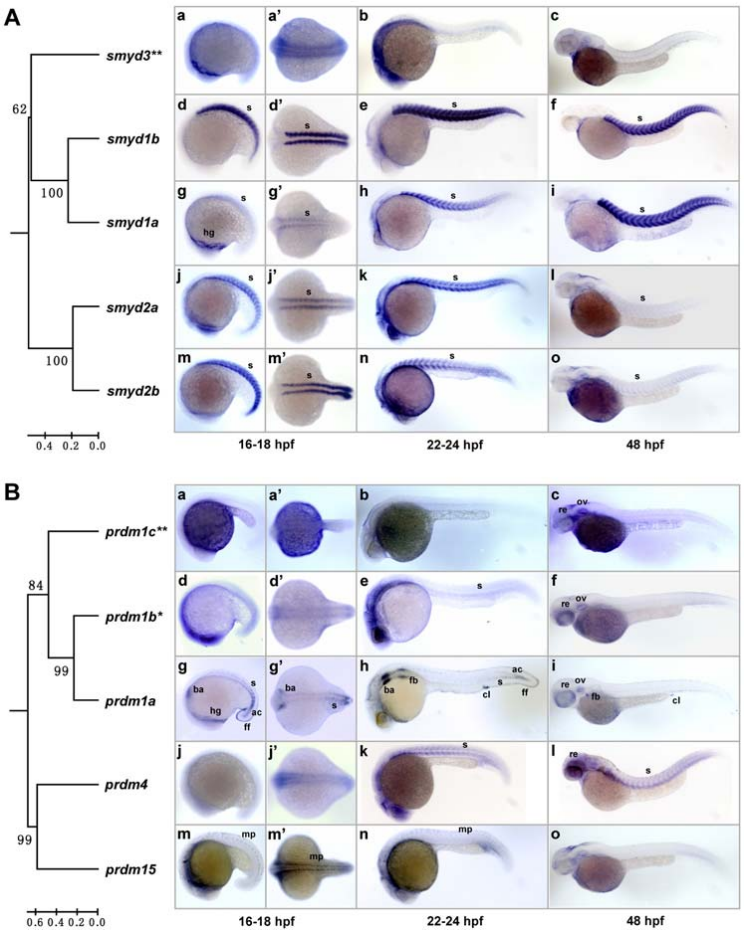
Somite/muscle-expressed SET domain genes and their evolutionary relationships. The phylogenetic relationships of the genes were indicated with the trees constructed based on the SET domains of the encoded proteins and rooted with zebrafish Smyd4 and Prdm14 proteins as outgroups, respectively. Lateral views (anterior to the left) of embryos at 16–18 hpf (a, d, g, j and m), 22–24 hpf (b, e, h, k and n) and 48 hpf (c, f, i, l and o) are presented. (a', d', g', j' and m') Dorsal views of the embryos in a, d, g, j and m. (A) Zebrafish *smyd1a*, *smyd1b*, *smyd2a* and *smyd2b* genes show somite/muscle-specific expression patterns and form a close paralog group with the *smyd3* gene (double asterisks), which shows a ubiquitous expression pattern (a–c). Note the relatively low expressions of *smyd1a* at early stage (18 hpf; g) and *smyd2a* and *smyd2b* at late stage (48 hpf; l and o). (B) Expression patterns of the second paralog group. *prdm1a* is specifically expressed in anterior somites and adaxial cells at 18 hpf (g and g') and 24 hpf (h). Besides, it is also expressed in hatching gland (g), branchial arch, fin fold (g, g' and h), fin buds, cloaca (h and i) and retina (i). *prdm1b* (asterisk) is highly expressed in somites at 24 hpf (e) and in retina at 48 hpf (f). *prdm1c* (double asterisks) is ubiquitously expressed (a–c). *prdm4* is highly expressed in somites and retina (k and l). *prdm1*5 is expressed in muscle pioneer cells (m, m' and n). ac, adaxial cells; ba, branchial arch; cl, cloaca; fb, fin buds; ff, fin fold; hg, hatching gland; mp, muscle pioneer; re, retina; s, somite.

In this study, eight somite/muslce-expressed SET domain genes were identified to be significantly expressed in somites and muscles at certain stages (Figure 5A and B, right). By merging the information of their evolutionary relationships, these genes were clustered into two groups, which subsequently distribute into subfamilies IX and X, respectively (Figure 5A and B, left). To a degree, their relationships suggest that these genes were evolved from two ancestral genes, both of which may be related with somite/muscle development of the ancestral species.

The first cluster includes 2 pairs of closely related zebrafish lineage-specific paralogs *smyd1a*, *smyd1b*, *smyd2a* and *smyd2b* (Figure 5A), though another closely related gene *smyd3* (GenBank accession DQ851821) (Figure 5A, double asterisks) shows a ubiquitous expression. Among these genes, *smyd1b* show highest specificity in somites and muscle cells. It was first detected in adaxial cells and anterior somites at 12 hpf (data not shown), and then highly in the muscle cells at 18–72 hpf (Figure 5Ad–f). Additionally, it is also specifically expressed in heart primordium (12 hpf) and mature heart (24–72 hpf) (Figure 5Ae, f and close-up pictures not shown). Similarly, its close paralog *smyd1a* is also specifically expressed in muscle cells at 18–72 hpf (Figure 5Ag–i). However, we did not observe its expression in heart. In mammals, the *SMYD1* gene was originally isolated from mouse CD8-positive T cells and named as *Bop* (*CD8b opposite*) [60]. The mouse *Smyd1*/*Bop* gene is also strongly expressed in skeletal and heart muscle; studies with *Smyd1*/*Bop* knockout mouse demonstrated that it is essential for cardiogenesis [61]. In agreement with that, a recent study indicated that zebrafish *smyd1b* gene is required for skeletal and cardiac muscle contraction [62], suggesting a good conservation between zebrafish and mouse. Zebrafish *smyd2a* and *smyd2b* are also highly expressed in somites and muscle cells at 18–72 hpf (Figure 5Aj–o). Meanwhile, *smyd2a* was observed to be significantly, though weakly, expressed in heart primordium at 12 hpf (close-up pictures not shown). The muscle-expression of mammalian *SMYD2* gene has not been reported so far. However, recent biochemical and cellular studies indicate that mammalian SMYD2 protein is an H3K36-specific HMT [63], and surprisingly, that it is able to methylate p53 on the lysine 370 and thereby inhibit the tumor suppressing function of p53 [64]. Furthermore, SMYD3, a H3K4-specific HMT, is also implicated in multiple cancers [44], [65], [66]. Considering the close evolutionary relationship among *SMYD1*, *SMYD2* and *SMYD3* genes, we hypothesize that they may share some common ancient mechanisms. A supporting evidence of this hypothesis is that overexpression of *SMYD3* in HEK293 cells significantly upregulate *NKX2.5*, a key cardiogenetic regulator [44]. Thus, based on this hypothesis, it is interesting to determine the potentially common epigenetic mechanisms in cardiogenesis, myogenesis and tumorigenesis.

The second cluster includes *prdm1a* (GenBank accession DQ851839), *prdm1b* (GenBank accession DQ851840), *prdm15* (GenBank accession DQ851842) and *prdm4* (GenBank accession DQ851843). The somite-related expression of *prdm1a* is first detected in adaxial cells and anterior somites at 12 hpf, and at the same time, it is also specifically expressed in prechordal mesoderm and border of the neural plate (data not shown). During 18–24 hpf, *prdm1a* is consistently expressed in the posterior somites (Figure 5Bg–h). Additionally, *prdm1a* is also expressed in branchial arch and fin fold (18–24 hpf Figure 5Bg–h), fin buds and cloaca (24–48 hpf; Figure 5Bh and i) and retina (48 hpf; Figure 5Bi). The multi-tissue expression of *prdm1a* suggests that it may be involved in variety of developmental processes. Indeed, studies with both mouse and zebrafish models indicate its important roles in the development of lymphocytes [67], germ cells [68], epidermal cells [69], neurons [70] and muscle cells [71]. In contrast, the two close paralogs *prdm1b* and *prdm1c* (GenBank accession DQ851841) at least partially lost the specificities through evolution: while *prdm1b* (Figure 5B, asterisk) is relatively highly expressed in somites at 24 hpf and in retina at 48 hpf (Figure 5Be and f), *prdm1c* (Figure 5B, double asterisks) shows a more ubiquitous expression pattern (Figure 5Ba–c). Within this cluster, *prdm4* is highly expressed in somites and retina at 24–48 hpf (Figure 5Bk and l), while *prdm1*5 is expressed in muscle pioneer cells (a type of non-migratory adaxial cells) at 18–24 hpf (Figure 5Bm, m' and n). Although the potential functions of *prdm4* and *prdm15* are still unclear, their particular expression patterns suggest that they may play a role in myogenesis.

### Nervous system-expressed SET domain genes

**Figure 6:**
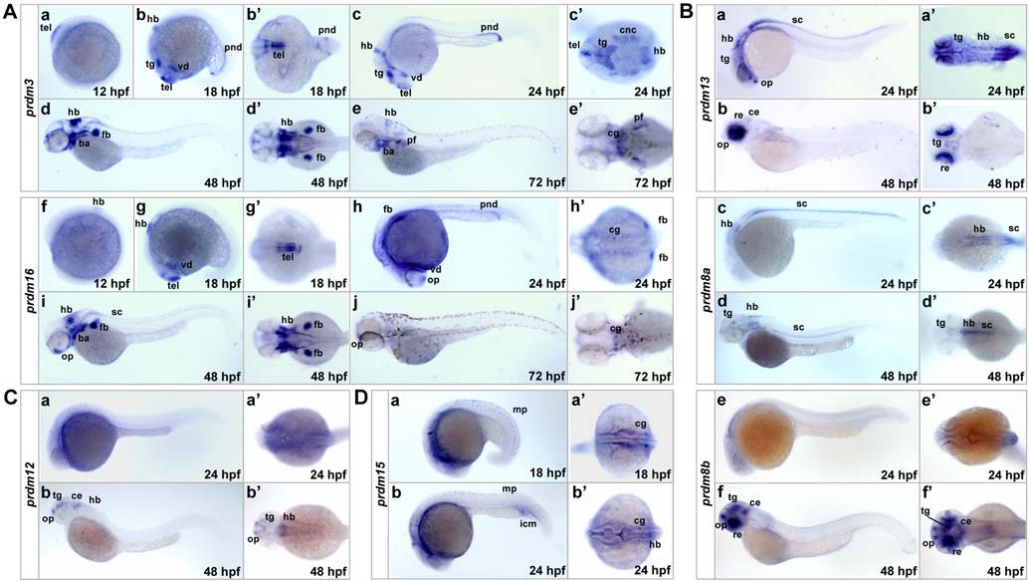
Nervous system-expressed SET domain genes. (A) Expression patterns of closely related *prdm3* and *prdm16*. (a–j) Lateral views (anterior to the left) of embryos at 12, 18, 24, 48 and 72 hpf. (b' and g') Ventral views of the embryos in b and g. (c'–e' and h'–j') Dorsal views of the embryos in c–e and h–j. Note the partially overlapping expression of *prdm3* and *prdm16*. (B) Expression patterns of *prdm13*, *prdm8a* and *prdm8b.* (a–f) Lateral views (anterior to the left) of embryos at 24 and 48 hpf. (a'–f') Dorsal views of the embryos in a–f. Note that the expression of *prdm8a* is mostly restricted in hindbrain and spinal chord (c, d and c', d'), whereas that of *prdm8b* is restricted in olfactory placode, tegmentum, cerebellum and retina (f and f'). (C) Expression pattern of *prdm12*. (a and b) Lateral views (anterior to the left) of embryos at 18, 24 hpf. (a' and b') Dorsal views of the embryos in a and b. At 48 hpf, prdm12 is expressed in olfactory placode, tegmentum, cerebellum and hindbrain. (D) Expression pattern of *prdm15*. (a and b) Lateral views (anterior to the left) of embryos at 18, 22 hpf. (a' and b') Dorsal views of the embryos in a and b. Note that *prdm15* is expressed in cranial ganglia neurons (a' and b') as well as in muscle pioneer cells and intermediate cell mass (a and b). ba, branchial arches; ce, cerebellum; cg, cranial ganglia; cnc, cranial neural crest; fb, fin buds; hb, hindbrain; icm, intermediate cell mass; mp, muscle pioneer; op, olfactory placode; pnd, pronephric duct; re, retina; sc, spinal chord; tel, telencephalon; tg, tegmentum; vd, ventral diencephalons.

Seven nervous system-expressed SET domain genes were identified by WISH analyses. The closely related *prdm3* (GenBank accession DQ851828) and *prdm16* (GenBank accession DQ851827) are expressed in a partially overlapping pattern (Figure 6A). The expression of *prdm3* is first detected in telencephalon at 12 hpf (Figure 6Aa) and then extends to tegmentum, ventral diencephalons and hindbrain at 18 and 24 hpf (Figure 6Ab and c). In addition, highly specific expression of *prdm3* in pronephric duct is apparent at 18 and 24 hpf (Figure 6Ab and c). At 48 and 72 hpf, *prdm3* is additionally expressed in branchial arches and pectoral fin buds (Figure 6Ad and e). In contras, *prdm16* is firstly detected in hindbrain rather than telencephalon (12 hpf; Figure 6Af). The fin buds-expression of *prdm16* appears earlier than that of *prdm3*, whereas the pronephric duct-expression of *prdm16* is not as specific as that of *prdm3* (Figure 6Ac and h). From 24 hpf to 72 hpf, the olfactory placode-expression of *prdm16* is relatively high (Figure 6Ah–j).

The closely related zebrafish *prdm13* (GenBank accession DQ851835), *prdm8a* (GenBank accession DQ851834) and *prdm8b* (GenBank accession DQ851833) are orthologous to human *PRDM13* and *PRDM8* genes, respectively (Figure 1A). WISH analysis indicates that they are specifically expressed in central nervous system and eyes (Figure 6B). Interestingly, although *prdm8a* and *prdm8b* display almost different expression patterns, combining the expression of both two genes highly resembles that of *prdm13*. In detail, *prdm13* is expressed in olfactory placode, tegmentum, hindbrain and spinal chord at 24 hpf (Figure 6Ba and a') and in retina, olfactory placode and tegmentum at 48 hpf (Figure 6Bb and b'). In contrast, *prdm8a* is expressed in hindbrain and spinal chord at 24 and 48 hpf (Figure 6Bc, c' and d, d'), whereas *prdm8b* is expressed in olfactory placode, tegmentum, cerebellum and retina at 48 hpf (Figure 6Bf and f'). According to their phylogenetic relationship, *prdm8a* and *prdm8b* are likely derived from an ancestral *prdm8* gene that is most closely related with prdm13 in function and expression. Thus, their distinct expression patterns of *prdm8a* and *prdm8b* are thought to reflect a subfunctionalization [72], by which *prdm8a* and *prdm8b* partition the different functions of the multifunctional ancestral *prdm8* gene. These observations would be helpful for understanding the function of these genes in the context of evolution. Meanwhile, we also discovered that *prdm12* (GenBank accession DQ851836) is restrictedly expressed in olfactory placode, tegmentum, cerebellum and hindbrain at 48 hpf (Figure 6Cb and b'), though it is ubiquitously and weakly expressed at 24 hpf (Figure 6Ca and a'). *prdm15* is observed to be expressed in cranial ganglia neurons (Figure 6Da' and b') as well as in muscle pioneer cells and intermediate cell mass (a and b) at 18 and 22 hpf. Taken together, these results provide major implications of involvement of these SET domain genes in neural development and have special reference to further studying their biological functions.

## Discussion

One of the objectives of this study is to comprehensively and non-redundantly identify all of zebrafish SET domain genes on a whole-genome scale, and subsequently to obtain a global view of this gene family in the context of evolution and development. To achieve this goal, on the one hand, we employed the zebrafish whole-genome shotgun trace database, which comprising a large amount of short reads with a coverage of >5×. This approach minimized the possibility of missing data, compared with the using of cDNA/EST database or the assembled genomic database. On the other hand, we effectively removed the redundancies of the retrieved putative SET domain genes of zebrafish, as well as those of other species, by carrying out sequence alignment, exon/intron structural analysis, phylogenetic analysis and chromosomal localization. Our analyses demonstrate that there are obvious redundancies in some domain databases (e.g. the SMART and Pfam databases account the number of human SET domain proteins as 79 and 105, respectively). These redundancies likely led to overestimates of the numbers of SET domain genes of various species in some previous literatures. Apart from the computer-based sequence analyses, we also directly cloned the 58 zebrafish SET domain genes in certain fragments by RT-PCR and determined their expression patterns by WISH analysis, further supporting the existence and expression of these genes.

The identification of zebrafish SET domain genes allows defining the relationship among the vertebrate SET domain genes and outlining an evolutionary history of this gene family in a wide range of species from yeast to human. First of all, our analyses indicate that the vertebrate SET domain genes can be divided into 10 subfamilies, which is supported by both the phylogeny of the SET domains (Figure 1A) and the similarities of the domain architectures (Figure S2). Notably, however, several precedent reports defined four main subfamilies of SET domain proteins, namely SET1 subfamily (including HRX and E(Z) groups), SET2 subfamily (also known as ASH1 subfamily), SUV39 subfamily and RIZ subfamily [73], [74]. Comparing our results with these studies showed that the discrepancy was largely due to a number of newly identified vertebrate SET domain proteins (e.g. SETMAR, SETD5, SETD6, SETD7, SETD8, etc.) that were included in our analyses. This comparison suggests that a genome-wide analysis favors an unbiased global view of a big gene family.

Second, we identified the zebrafish lineage- and human lineage-specific SET domain genes and determined their origins through evolution, which explores the diversities between zebrafish and human in terms of SET domain-related epigenetic regulation. While the twelve pairs of zebrafish lineage-specific paralogs were generated from the WGD in teleost, the two pairs of human lineage-specific paralogs, namely *SUV39H1*/*SUV39H2* and *PRDM7*/*PRDM9*, were raised from two different duplication events through evolution. It is interesting to note that mouse *Suv39h2* and *Prdm9* (also known as *Meisetz*) genes have been implicated in germ cell development. In particular, both two genes are specifically expressed in adult testis [75], [76], though *Suv39h2* has been detected in a rather uniform expression at embryonic stage [75]. Importantly, *Prdm9*/*Meisetz* knockout mice are viable but sterile, suggesting that the SET domain-mediated epigenetic regulation is crucial for germ cell development and reproduction [76]. The relatively recent gene duplication and fixation events of these master genes suggest that the mechanisms of epigenetic regulation in reproduction may have subtle divergences among different vertebrates. Thus, on the strength of an evolutionary view of these genes, comparative study of these processes among different models (e.g. zebrafish, mouse and human) should contribute to deep understanding the mechanisms.

Third, the identification of COGs of the SET domain genes ranging from yeast to human provides a fundamental framework, by which one can integrate the large amount of information about these SET domain genes in various species obtained from the structural and functional studies, and subsequently predict the function of any gene member that has not been well studied. Furthermore, an application of these results is to explain the origins of the site specificities of SET domain HMTs. Generally, there are at least two possible explanations to the origins of the so far defined multiple site specificities of SET domain HMTs: 1) all the SET domains with a same specificity originate from a single ancestor; or 2) the SET domain is able to acquire a new specificity during evolution. As shown in Figure 4, the COGs in subfamilies I and V are corresponding to single specificities H3K9 (note that the amino acid context of H3K9 resembles that of K27, which likely leads to the dual-specificities of EHMT1 and EHMT2 [25]) and H3K4, respectively. However, the COGs in subfamilies III and IX have been indicated to possess different specificities. Particularly, human *NSD1*, *WHSC1* and *WHSC1L1* genes share high homology (Figure S4) and are obviously co-orthologous to fruit fly *Mes-4* gene. However, NSD1 and WHSC1L1 HMTs have been proven to carry totally different specificities [23], [77], suggesting that at least one of them has changed its specificity during evolution, which support the second possibility described above. This kind of events may result in species-specific mechanisms of writing and reading the histone code. Furthermore, if this assumption is true, a SET domain HMT may also evolve to acquire a novel specificity. Taken together, these observations and analyses would contribute to the explanation of the recently identified novel and/or species-specific histone methylation patterns [12].

A comprehensive developmental expression profile of the whole family of SET domain genes should augment the value of the evolutionary perspectives of these genes, and more directly, provide useful information for functional studies of certain zebrafish SET domain genes. Gene-specific knockdown strategies, especially by means of Morpholino oligos, have been widely used to effectively silence both maternal and zygotic mRNA in zebrafish [78]. Among the zebrafish SET domain genes, fifteen maternally expressed ones, which may largely control the programming of histone modification states during very early development, has been identified. Taking advantages of the external development and optical clarity of zebrafish embryos, we showed that the histone modification can be easily detected by fluorescent staining (Figure S1). Meanwhile, by means of Western blot, we observed that the histone is cleaved at certain stage (6–12 hpf) of zebrafish embryogenesis (unpublished data), which is highly consistent with E. M. Duncan's finding of histone proteolysis during ES cell differentiation (Duncan et al., an abstract of the 2007 Keystone Symposia: Epigenetics. Breckenridge, Colorado, April 11–16, 2007). Taken together, our data support that the zebrafish embryos are particularly amenable to studies of the roles of histone modification during early development.

Thirteen tissue-specific zebrafish SET domain genes, which may play a relatively specific role in organogenesis, were identified in this study. These genes are more beneficial for functional analysis, because the effects of Morpholino-mediated knockdown of each gene should be located in certain tissues and lead to a specific phenotype. Importantly, the human orthologs of several these genes have been implicated in tumorigenesis, thus functional characterization of these genes is of great importance. For example, the mouse *Prdm3* gene was firstly identified as a common locus of retroviral integration in myeloid leukemia and thereby name as *ecotropic viral integration site 1* (*evi1*) [79]. The retroviral integration within this gene is implicated in the alteration of self-renewal or survival of hematopoietic stem cells [80]. Furthermore, human *PRDM3*/*EVI1* gene is frequently involved in chromosomal translocation with variety of partner genes, including *AML1*/*RUNX1*, leading to myelodysplasia and acute myeloid leukemia [81]. The *PRDM16* gene, also named as *MEL1* for *MDS1-EVI1-like gene 1*, is also involved in leukemogenesis via chromosomal translocation [82]. The herein characterized high conservation of these genes between zebrafish and mammals suggest zebrafish as a model to be applied to determine the mechanisms underlying tumorigenesis.

## Materials and Methods

### Data sources of genomic and cDNA sequences

Zebrafish whole-genome shotgun trances and the assemblies were obtained from the Ensembl Zebrafish Genome Server (ftp://ftp.ensembl.org/pub/traces/danio_rerio/fasta). The cDNA and EST sequence data of zebrafish and other species were obtained from NCBI (http://www.ncbi.nlm.nih.gov/). Some of zebrafish cDNA sequence data were obtained from our zebrafish kidney cDNA project described previously [34]. Amino acid sequences of SET domains of various species were obtained from SMART database (http://smart.embl-heidelberg.de/) and from NCBI by PSI-BLAST searching against the non-redundant protein database.

### Identification and cloning of zebrafish SET domain genes

Using the sequences of human and fruit fly SET domains as search queries, a TBLASTN analysis was performed against zebrafish genome shotgun trances database. The cut-off E value was set as 1e-5. The Pangea CAT3.5 program was used to cluster and align the resulting sequences. The GENSCAN program (http://genes.mit.edu/GENSCAN.html) was used to predict the exons. The resulting putative mRNA sequences were extended by *in silico* EST assembly and the encoded protein sequences were deduced. The predicted zebrafish genes and proteins were named after their closest human homologues. For a portion of predicted zebrafish genes, their full-length ORFs cannot be obtained. However, the almost completed ORFs consisting of predicted exons can be used to determine the evolutionary relationships and expression patterns. The zebrafish SET domain genes were cloned from RT-PCR products of zebrafish embryos or adults. In brief, pools of zebrafish embryos at 0.75, 2, 4, 6, 9, 12, 18, 24, 48, 72 and 120 hpf or 1-year-old male and female adults were homogenized and subject to total RNA isolation with TRIZOL Reagent (Invitrogen) followed by DNase I (Invitrogen) treatment. RT-PCR was performed with SuperScript II Reverse Transcriptase (Invitrogen), followed by PCR-amplification using gene-specific primers containing EcoR I and Xho I (or Sal I) restriction sites on each side. The products were excised with corresponding restriction endonucleases (New England BioLabs) and cloned into the pCS2^+^ vector between the EcoR I-Xho I sites. On the other hand, the SET domain genes found in our zebrafish kidney cDNA library constructed with pBK-CMV vector (Stratagene) were also picked out. The insert sequences of all these plasmids were confirmed by direct sequencing.

### Phylogenetic analysis and ortholog prediction

The amino acid sequences of SET domains were aligned with ClustalX 1.83 program [83]. The BLOSUM series matrix was used and the end gap separation option was turn on. The resulting alignments were manually modified using BioEdit program (http://www.mbio.ncsu.edu/BioEdit/bioedit.html). Based on the alignments, phylogenetic trees were constructed using the neighbor-joining method with 1000 bootstrap replicates using the MEGA 3.1 program. Pairwise-deletion option was used to handle gaps and missing data. A BLAST-based “reciprocal best hit” method, in combination with phylogenetic analysis and genomic structure comparison, was used to determine the orthologous relationships and to identify the COGs. The complete nucleotide database of zebrafish was built by combining the zebrafish whole-genome shotgun trace database, NCBI EST and mRNA databases, while the protein databases of other species were extracted from the NCBI GenBank and Reference Sequences. To identify the orthologs between human and zebrafish, for example, each predicted zebrafish SET domain gene was subject to BLASTX analysis against the human protein database, and the top matching hits were then subject to TBLASTN analysis against the complete zebrafish nucleotide database. Finally, the orthologous relationship was recognized when the best hits overlap with the original query.

### Exon/intron structure, chromosomal location and syntenic analyses

The exon/intron structures of human genes were taken from the annotation of genomic sequences in GenBank, whereas those of the zebrafish genes were determined by comparison of cDNA sequences with genomic contigs, in combination with GENSCAN prediction, peptide translation and also making reference to the “GT-AG” splicing rule. Some splicing sites were confirmed by RT-PCR and sequencing. Chromosome location and gene orders of zebrafish genes were obtained from the latest zebrafish whole-genome assembly Zv6 and zebrafish genome mapping information from the ZFIN website (http://zfin.org), and those of the genes of human and other species were obtained from the NCBI Map Viewer (http://www.ncbi.nlm.nih.gov/mapview/). To analyze syntenies, putative zebrafish genes were identified within a limited region (≤500 kb) of zebrafish genomic contigs containing a SET domain gene, by means of GENSCAN analysis and EST alignment. Then these genes were subject to ortholog identification against human non-redundant protein database and the identified proteins were linked to the NCBI Map Viewer. The chromosomal locations of these ortholog pairs were drawn to scale along human and zebrafish chromosomes, thus revealing conserved syntenies for the SET domain genes and the neighboring genes between human and zebrafish.

### Zebrafish maintenance and embryo preparation

The zebrafish were maintained and staged as described previously [55]. Embryos raised to time points beyond 24 hpf were treated with 0.003% phenylthiourea to prevent melanization. Embryos at 18, 24, 48, 72 and 120 hpf were removed from chorions with 0.001% pronase (those of 0.75, 2, 4, 6, 9 and 12 hpf were dechorionated manually) and fixed overnight in 4% paraformaldehyde (Sigma) at 4°C. Fixed embryos were washed in PBST (phosphate-buffered saline supplemented with 0.1% Tween-20) and dehydrated in graded PBST/methanol solutions (3∶1, 1∶1, 1∶3) for 10 min each and stored in absolute methanol at −20°C.

### Immunofluorescence

The fixed embryos were rehydrated in graded PBST/methanol solutions (1∶3, 1∶1, 3∶1) for 10 min each, followed by PBST rinse twice for 10 min each at room temperature (RT). The embryos were permeabilized with 0.5% Triton X-100 in PBST for 15 min and rinsed twice with PBST for 10 min each at 4°C. After blocking in PBST containing 1% bovine serum albumin (BSA) for 1 hour at 4°C, the embryos were incubated with primary antibodies (see the legends of Figure S1) in 1% BSA/PBST overnight, washed with PBST three times for 30 min each, followed by incubation with Rhodamine-conjugated anti-mouse IgM (Pierce) or Alexa Fluor 488-conjugated anti-rabbit IgG (Molecular Probes) in 1% BSA/PBST for 1 hour at 4°C and washing with PBST three times for 30 min each. The embryos were photographed using a Nikon SMZ1500 Zoom Stereomicroscope.

### Whole-mount *in situ* hybridization

Antisense RNA probes were synthesized with T3 digoxigenin RNA Labeling Kit (Roche) from the cDNAs in the pCS2+ vector and purified with NucAway Spin Columns (Ambion). The fixed embryos were rehydrated. Embryos beyond 24 hpf were permeabilized with proteinase K solution (100 μg/ml; Sigma) at RT for 20-30 min, rinsed in PBST twice, and refixed in 4% paraformaldehyde at RT for 30 min. Ten to 15 embryos from each time points were combined and hybridized with digoxigenin-labeled antisense RNA probes at 68°C. After extensive washing, the probes were detected with Anti-digoxigenin-AP Fab fragments (1∶5000; Roche), followed by staining with BCIP/NBP Alkaline Phosphatase Substrate (VECTOR laboratories). The embryos were mounted in 30% methylcellulose/PBST and photographed using the Nikon SMZ1500 Zoom Stereomicroscope.

## Supporting Information

Figure S1 Immunofluorescent analyses of RNA polymerase II phosphorylation and histone H3K36 methylation in zebrafish embryos. Zebrafish embryos at different stages were subject to immunofluorescent staining to detect the unmodified pol II (A) and hyperphosphorylated pol II (B and C), H3K36 monomethylation (D), dimethylation (E) and trimethylation (F). Immunofluorescent staining of histone H3 (G) was used as a positive control. While the staining of histone H3 in nuclei is consistently detected (G), the staining of H3K36 methylation cannot be detected until 64-cell stage (D–F). The inset panels show the magnified views of detected staining in nuclei (arrow head). The unmodified, serine 2-phosphorylated and serine 5-phosphorylated pol II were probed with mouse monoclonal antibodies 8WG16, H5 and H14 (Covance Research Products), respectively. H3K36 mono-, di- and trimethylation were probed with rabbit polyclonal antibodies ab9084 (ABcam), 07-274 (Upstate) and ab9050 (Abcam), respectively. Histone H3 was probed with rabbit polyclonal antibody ab1791 (Abcam).
(4.13 MB TIF)

Figure S2 Domain architectures of vertebrate SET domain proteins. The domain architectures of the full-length proteins (*middle*) were drawn based on the searches of the SMART database. The phylogenetic tree (*left*) was derived from Figure 1A by compressing subtrees according to the combined information of topology of the tree and the domain architectures. Note that several proteins are corresponding to each of the structures shown (*right*), despite little divergence in the spatial arrangement of the domains. Parentheses indicate a domain that not all members of a given group contain, whereas underlines indicate that the number of a domain is variable among the members. In subfamily I, IV and VI, the members were divided into several groups according to the divergences in domain architectures. Notably, these results of domain architecture analysis of the full-length proteins are highly consistent with the phylogenetic analysis of the SET domains alone.
(2.53 MB TIF)

Figure S3 Conserved syntenies among zebrafish lineage-specific SET domain gene pairs and their human counterparts. The SET domain genes are indicated in *red* while the neighboring genes in *black*. Chromosome numbers of human (*Hs*) and zebrafish (*Dr*) are shown. The chromosomal locations of human genes are shown in parentheses after the gene names. Distances between genes on a single chromosome are shown to scale, and the compared chromosomes are scaled to equivalent lengths. Lines between the compared chromosomes connect positions of orthologous gene pairs in the two species. Of note, most zebrafish genes, only with exception of *mll4a* and *prdm1b* genes, show obviously conserved syntenic relationship with their human counterparts. The zebrafish *prdm1c* gene shows conserved synteny with human *PRDM1* gene, although these two genes have only a moderate similarity in amino acid sequence.
(2.34 MB TIF)

Figure S4 Phylogenetic analysis of SET domain proteins ranging from yeast to human. Unrooted neighbor-joining tree was constructed based on the alignment of the amino acid sequences of the SET domain proteins of human (*red*), zebrafish (*blue*), *Drosophila* (*purple*), *C. elegans* (*pink*), *S. pombe* (*green*) and *S. cerevisiae* (*olive*). Note that the 10 subfamilies defined with vertebrate SET domain genes (Figure 1A) are also clearly distinguishable, as denoted with light blue curves.
(3.44 MB TIF)

Figure S5 Representative examples of ubiquitously expressed SET domain genes with relatively higher expression in certain tissues. Lateral views (anterior to the left) of embryos at 18 hpf (a, c, e and g) and 24 hpf (b, d, f and h) are presented. Note that *whsc1* (a and b) and *ezh2* (c and d) are highly expressed in the central nervous system, whereas *ezh2* (c and d), *setd2* (e and f) and *mll5* (g and h) are highly expressed in intermediate cell mass of mesoderm. cns, central nervous system; icm, intermediate cell mass.
(2.90 MB TIF)

Figure S6 Expression of SET domain genes before the onset of zygote gene transcription. WISH analyses of 58 zebrafish SET domain genes at 0.75, 2 and 4 hpf were representatively shown.
(5.11 MB TIF)

Table S1 SET domain genes that were analyzed in this study. Note that we named the SET domain genes according to the current nomenclature in the Entrez Gene. Meanwhile, some other frequently used names of these genes were also listed as “Other Aliases”. *Drosophila proteins msta-A and msta-B are encoded by two alternative splicing isoforms of msta gene, and notably, they contain different SET domains.
(0.21 MB PDF)

Table S2 Comparison of the expression patterns of zebrafish genes with their mouse counterparts revealed by mRNA in situ hybridization assays
(0.12 MB PDF)
