# Supplementary figures and images for: Genome-Wide Survey and Developmental Expression Mapping of Zebrafish SET Domain-Containing Genes
Source: PLoS One. 2008 Jan 30;3(1):e1499. doi: 10.1371/journal.pone.0001499 (PMC2200798; doi:10.1371/journal.pone.0001499)

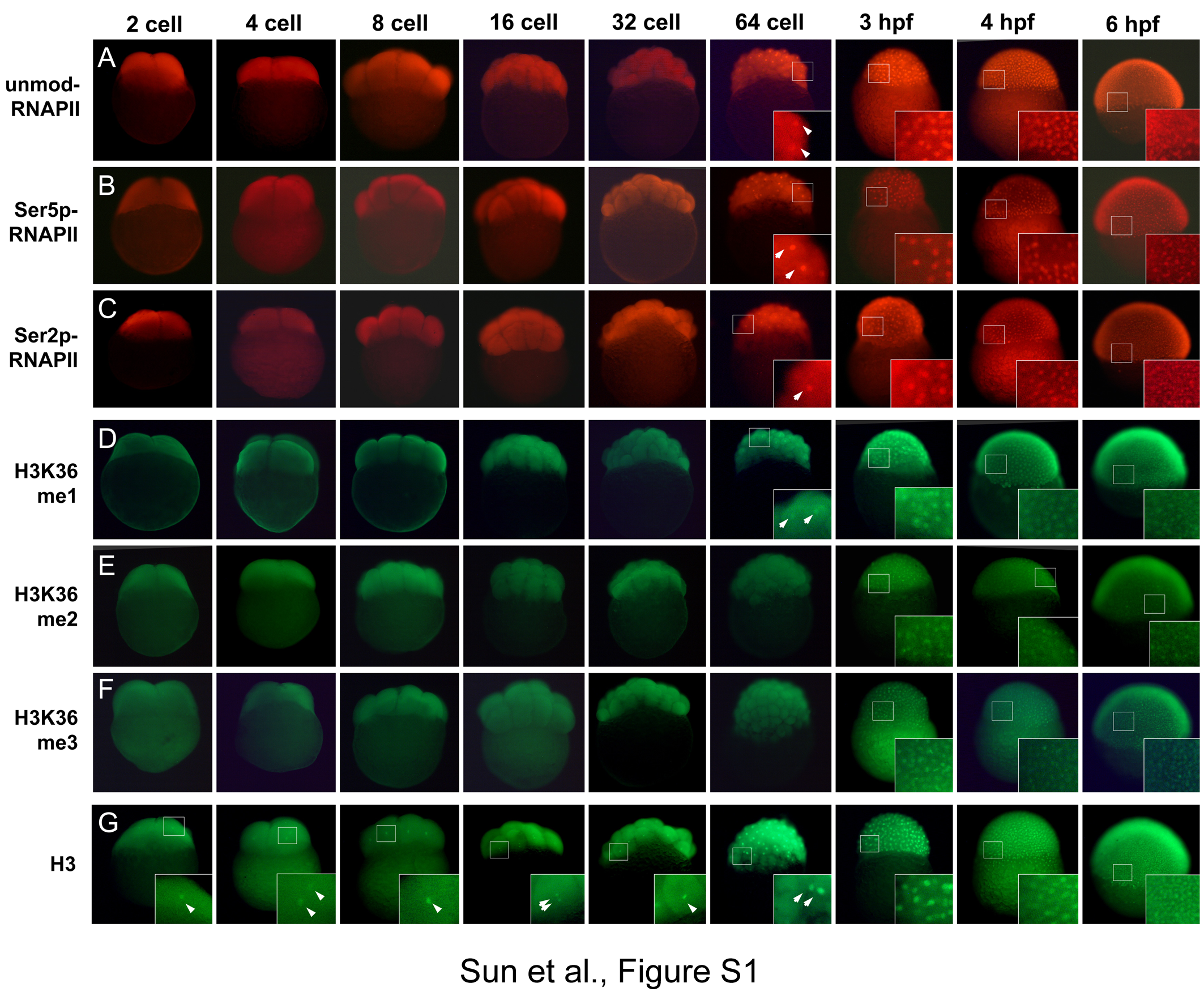

Supplement: Figure S1 — Immunofluorescent analyses of RNA polymerase II phosphorylation and histone H3K36 methylation in zebrafish embryos. Zebrafish embryos at different stages were subject to immunofluorescent staining to detect the unmodified pol II (A) and hyperphosphorylated pol II (B and C), H3K36 monomethylation (D), dimethylation (E) and trimethylation (F). Immunofluorescent staining of histone H3 (G) was used as a positive control. While the staining of histone H3 in nuclei is consistently detected (G), the staining of H3K36 methylation cannot be detected until 64-cell stage (D–F). The inset panels show the magnified views of detected staining in nuclei (arrow head). The unmodified, serine 2-phosphorylated and serine 5-phosphorylated pol II were probed with mouse monoclonal antibodies 8WG16, H5 and H14 (Covance Research Products), respectively. H3K36 mono-, di- and trimethylation were probed with rabbit polyclonal antibodies ab9084 (ABcam), 07-274 (Upstate) and ab9050 (Abcam), respectively. Histone H3 was probed with rabbit polyclonal antibody ab1791 (Abcam). (4.13 MB TIF) [file pone.0001499.s001.tif]

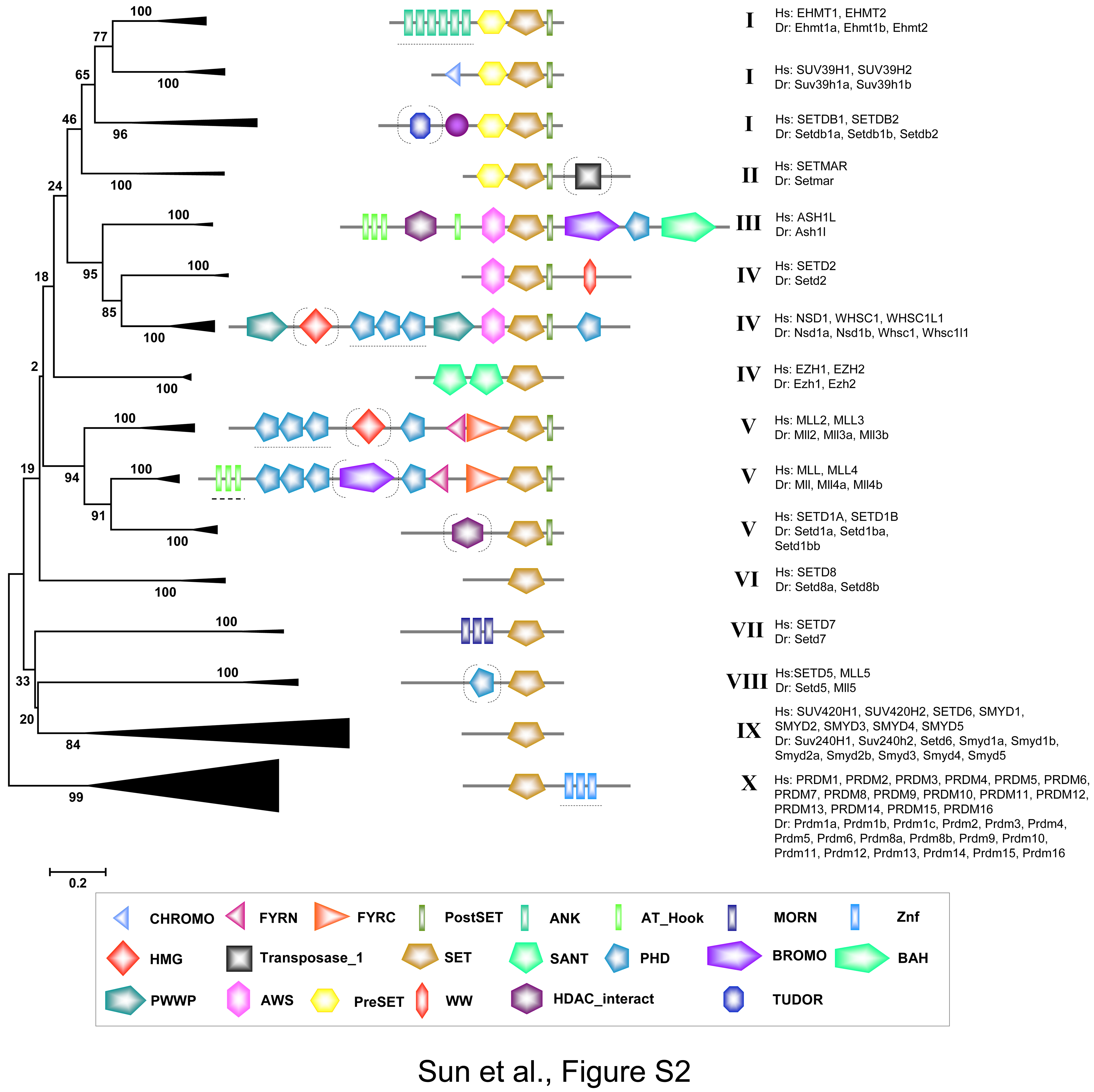

Supplement: Figure S2 — Domain architectures of vertebrate SET domain proteins. The domain architectures of the full-length proteins (middle) were drawn based on the searches of the SMART database. The phylogenetic tree (left) was derived from Figure 1A by compressing subtrees according to the combined information of topology of the tree and the domain architectures. Note that several proteins are corresponding to each of the structures shown (right), despite little divergence in the spatial arrangement of the domains. Parentheses indicate a domain that not all members of a given group contain, whereas underlines indicate that the number of a domain is variable among the members. In subfamily I, IV and VI, the members were divided into several groups according to the divergences in domain architectures. Notably, these results of domain architecture analysis of the full-length proteins are highly consistent with the phylogenetic analysis of the SET domains alone. (2.53 MB TIF) [file pone.0001499.s002.tif]

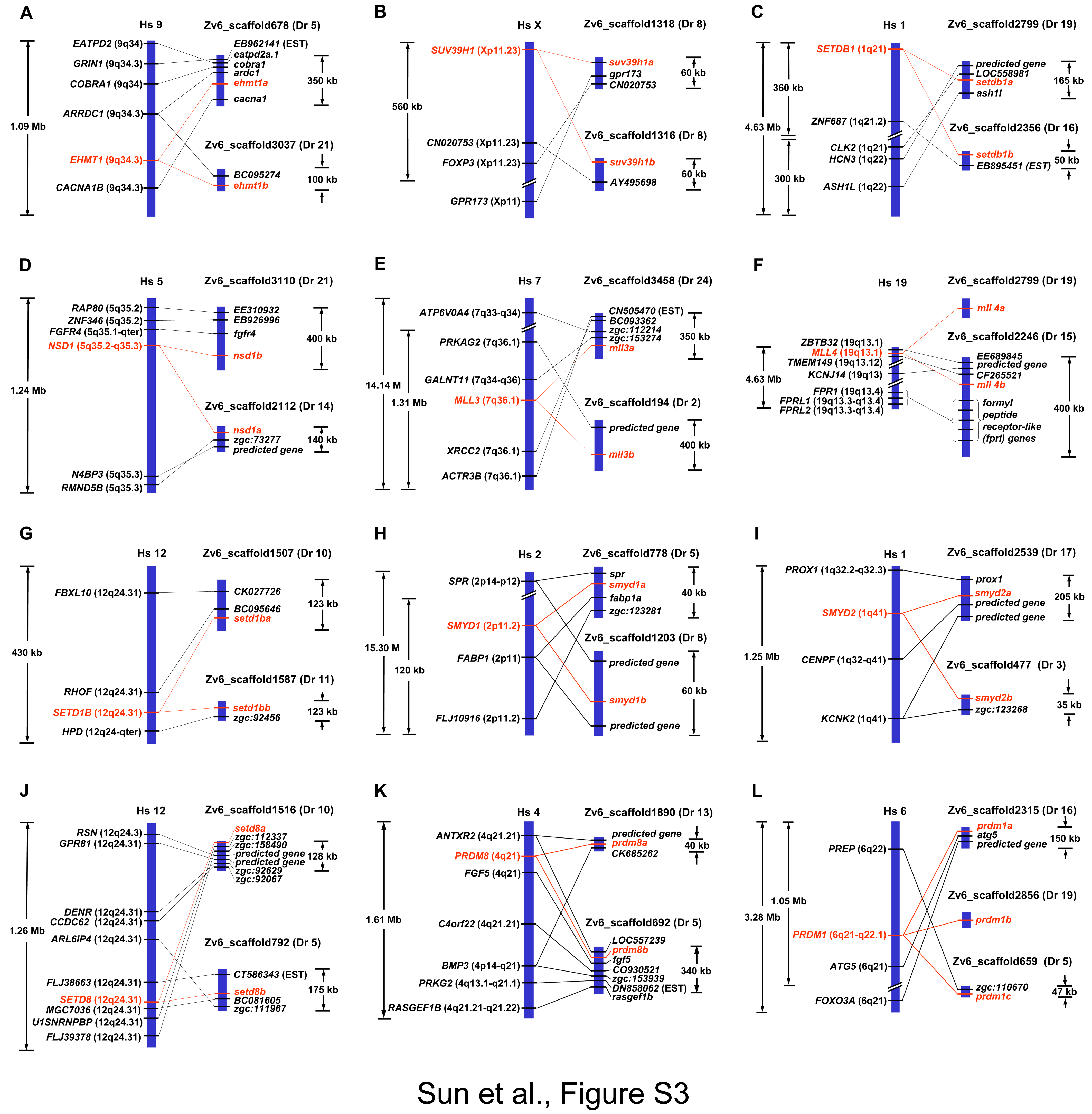

Supplement: Figure S3 — Conserved syntenies among zebrafish lineage-specific SET domain gene pairs and their human counterparts. The SET domain genes are indicated in red while the neighboring genes in black. Chromosome numbers of human (Hs) and zebrafish (Dr) are shown. The chromosomal locations of human genes are shown in parentheses after the gene names. Distances between genes on a single chromosome are shown to scale, and the compared chromosomes are scaled to equivalent lengths. Lines between the compared chromosomes connect positions of orthologous gene pairs in the two species. Of note, most zebrafish genes, only with exception of mll4a and prdm1b genes, show obviously conserved syntenic relationship with their human counterparts. The zebrafish prdm1c gene shows conserved synteny with human PRDM1 gene, although these two genes have only a moderate similarity in amino acid sequence. (2.34 MB TIF) [file pone.0001499.s003.tif]

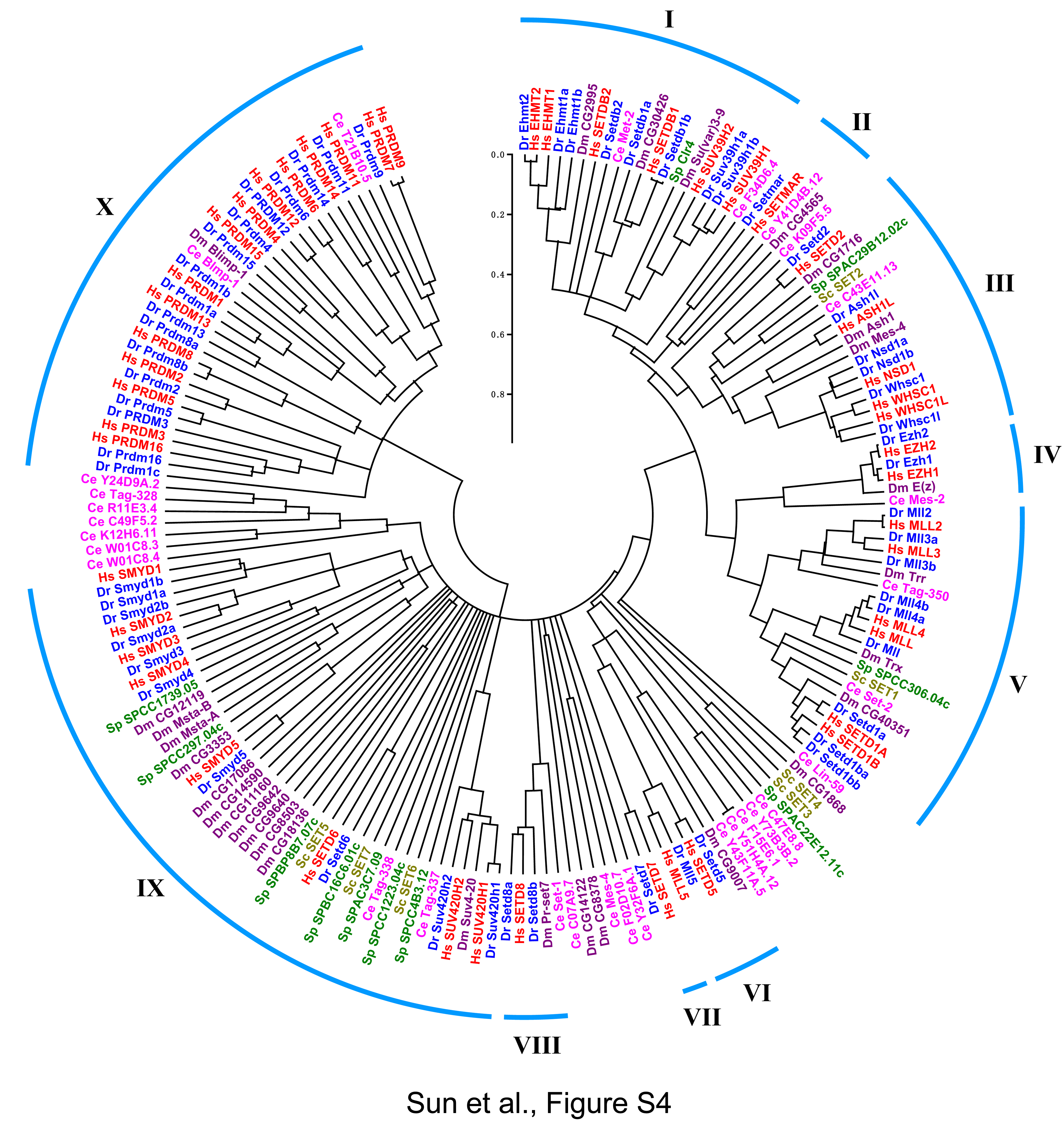

Supplement: Figure S4 — Phylogenetic analysis of SET domain proteins ranging from yeast to human. Unrooted neighbor-joining tree was constructed based on the alignment of the amino acid sequences of the SET domain proteins of human (red), zebrafish (blue), Drosophila (purple), C. elegans (pink), S. pombe (green) and S. cerevisiae (olive). Note that the 10 subfamilies defined with vertebrate SET domain genes (Figure 1A) are also clearly distinguishable, as denoted with light blue curves. (3.44 MB TIF) [file pone.0001499.s004.tif]

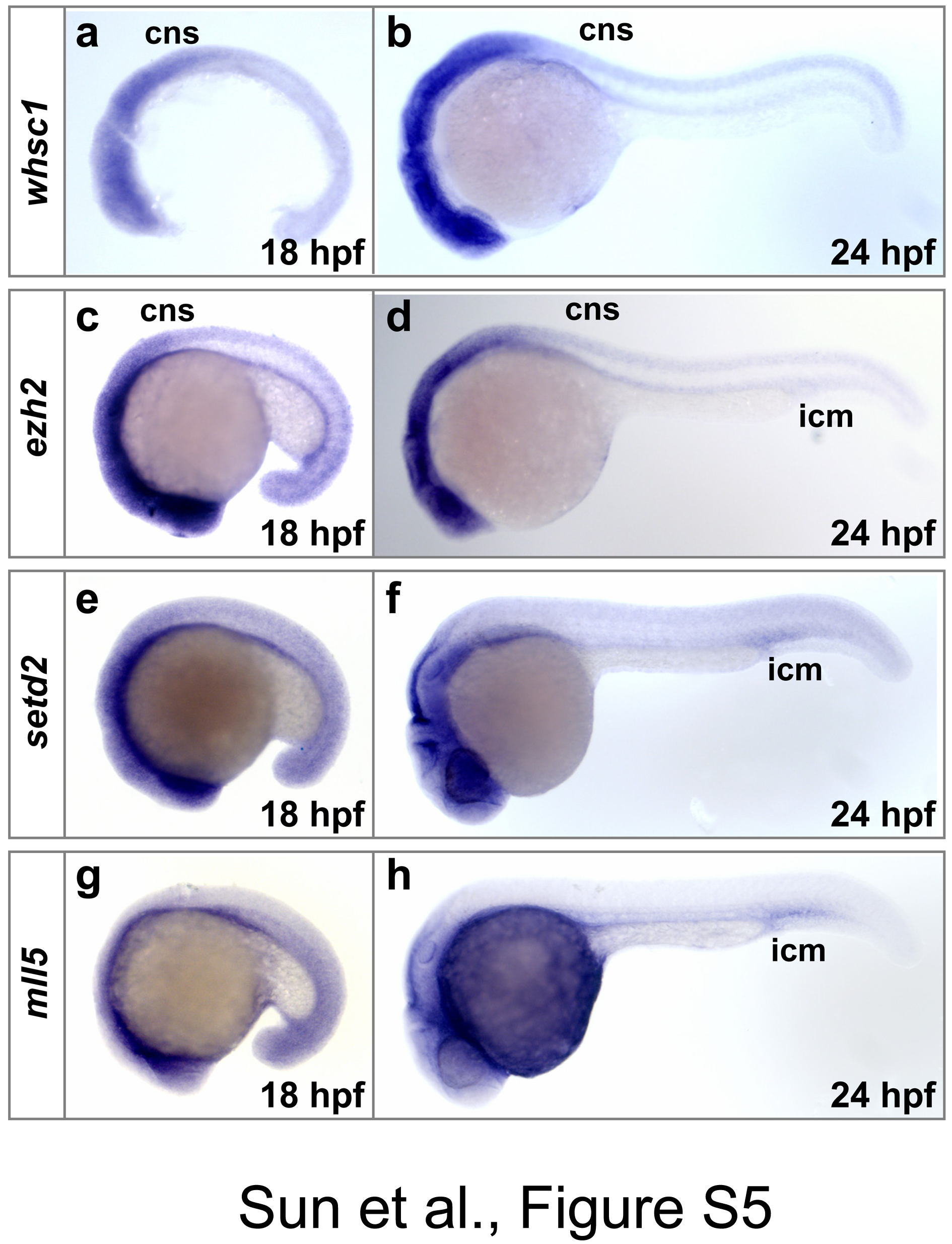

Supplement: Figure S5 — Representative examples of ubiquitously expressed SET domain genes with relatively higher expression in certain tissues. Lateral views (anterior to the left) of embryos at 18 hpf (a, c, e and g) and 24 hpf (b, d, f and h) are presented. Note that whsc1 (a and b) and ezh2 (c and d) are highly expressed in the central nervous system, whereas ezh2 (c and d), setd2 (e and f) and mll5 (g and h) are highly expressed in intermediate cell mass of mesoderm. cns, central nervous system; icm, intermediate cell mass. (2.90 MB TIF) [file pone.0001499.s005.tif]

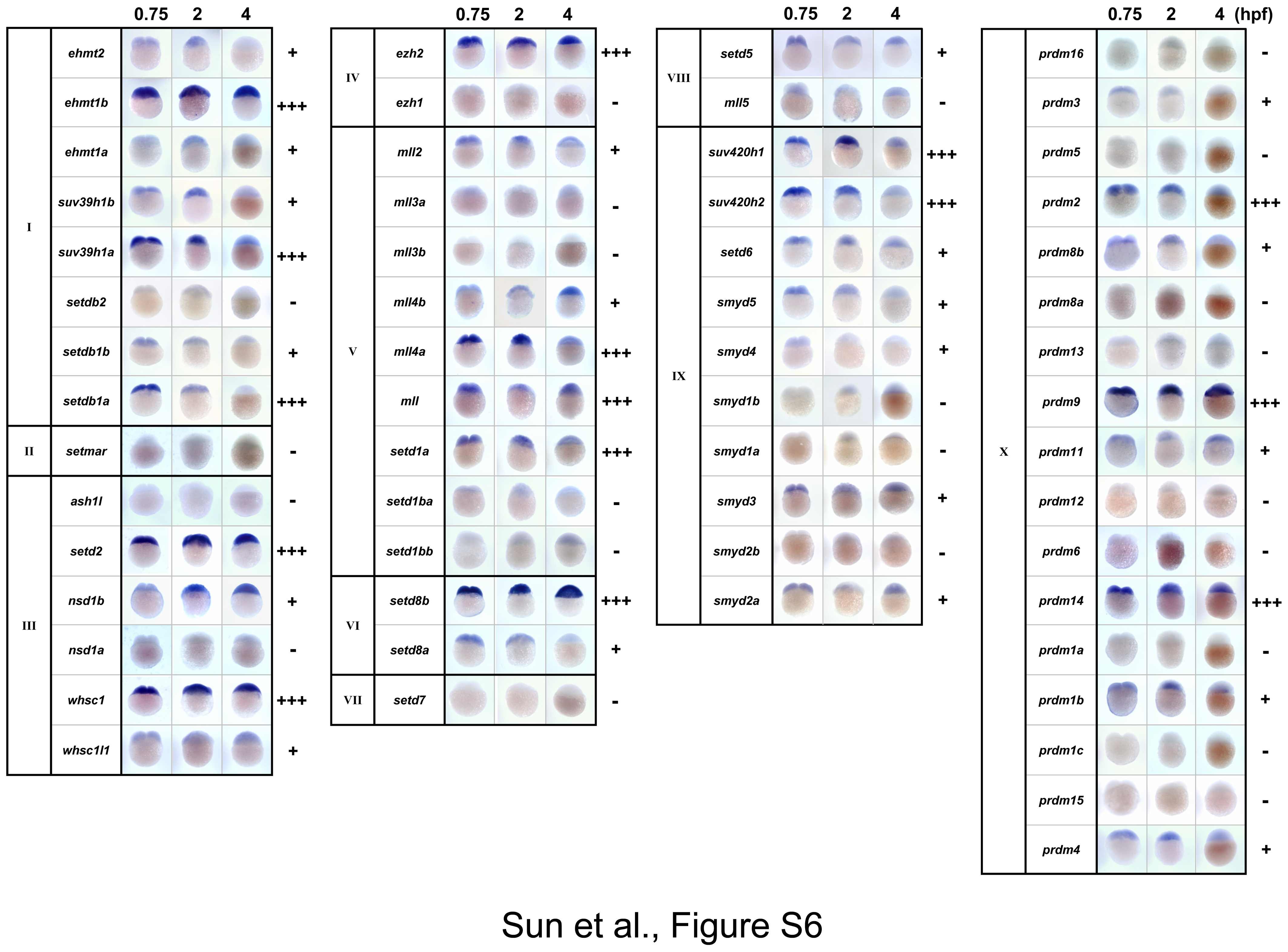

Supplement: Figure S6 — Expression of SET domain genes before the onset of zygote gene transcription. WISH analyses of 58 zebrafish SET domain genes at 0.75, 2 and 4 hpf were representatively shown. (5.11 MB TIF) [file pone.0001499.s006.tif]
