# Supplementary material for: Genome-Wide Survey and Developmental Expression Mapping of Zebrafish SET Domain-Containing Genes
Source: PLoS One. 2008 Jan 30;3(1):e1499. doi: 10.1371/journal.pone.0001499 (PMC2200798; doi:10.1371/journal.pone.0001499)
Supplement: Table S1 — SET domain genes that were analyzed in this study. Note that we named the SET domain genes according to the current nomenclature in the Entrez Gene. Meanwhile, some other frequently used names of these genes were also listed as “Other Aliases”. *Drosophila proteins msta-A and msta-B are encoded by two alternative splicing isoforms of msta gene, and notably, they contain different SET domains. (0.21 MB PDF) [file pone.0001499.s007.pdf]

Table S1. SET domain genes that were analyzed in this study

Gene Name	Description	Other Aliases	GenBank Accession Number	Chromosomal Location	HMT activity	Reference (PMID)
Homo sapiens						
ASH1L	ash1 (absent, small, or homeotic)-like (Drosophila)	*huASH1*	NP_060959	1q22	n.a.	
EHMT1	euchromatic histone-lysine N-methyltransferase 1	*GLP; Eu-HMTase1*	NP_079033	9q34.3	H3K9	12004135
EHMT2	euchromatic histone-lysine N-methyltransferase 2	*G9A*	NP_006700	6p21.31	H3K9, K27	11316813
EZH1	enhancer of zeste homolog 1		NP_001982	17q21.1-q21.3	n.a.	
EZH2	enhancer of zeste homolog 2		NP_004447	7q35-q36	H3K27	12351676, 12435631
MLL	myeloid/lymphoid or mixed-lineage leukemia (trithorax homolog, Drosophila)	*ALL-1; HRX*	NP_005924	11q23	H3K4	12453418, 12453419
MLL2	myeloid/lymphoid or mixed-lineage leukemia 2	*ALR*	NP_003473	12q12-q14	H3K4	12482968, 14992727
MLL3	myeloid/lymphoid or mixed-lineage leukemia 3	*HALR*	NP_067053	7q36.1	H3K4	12482968
MLL4	myeloid/lymphoid or mixed-lineage leukemia 4	*HRX2; TRX2*	NP_055542	19q13.1	n.a.	
MLL5	myeloid/lymphoid or mixed-lineage leukemia 5		NP_061152	7q22.1	n.a.	
NSD1	nuclear receptor binding SET domain protein 1		NP_758859	5q35.2-q35.3	H3K36, H4K20	12805229
PRDM1	PR domain containing 1, with ZNF domain	*BLIMP1; PRDI-BF1*	NP_001189	6q21-q22.1	n.a.	
PRDM11	PR domain containing 11		NP_064614	11p11	n.a.	
PRDM12	PR domain containing 12		NP_067632	9q33-q34	n.a.	
PRDM13	PR domain containing 13		NP_067633	6q16-q21	n.a.	
PRDM14	PR domain containing 14		NP_078780	8p21-p12	n.a.	
PRDM15	PR domain containing 15		AAF78093	21q22.3	n.a.	
PRDM16	PR domain containing 16	*MEL1*	NP_071397	1p36.23-p33	n.a.	
PRDM2	PR domain containing 2, with ZNF domain	*RIZ*	NP_036363	1p36.21	H3K9	14633678
PRDM3	PR domain containing 3	*EVI-1; MDS1-EVI1*	AAB29907	3q24-q28	n.a.	
PRDM4	PR domain containing 4		NP_036538	12q23-q24.1	n.a.	
PRDM5	PR domain containing 5		NP_061169	4q25-q26	n.a.	
PRDM6	PR domain containing 6		AAF78078	5q23.2	n.a.	
PRDM7	PR domain containing 7		AAF78084	16q24.3	n.a.	
PRDM8	PR domain containing 8		NP_064611	4q21	n.a.	
PRDM9	PR domain containing 9		NP_064612	5p14	n.a.	
SETD1A	SET domain containing 1A	*Set1*	AAH27450	16p11.2	H3K4	12670868
SETD1B	SET domain containing 1B		BAA83028	12q24.31	n.a.	
SETD2	SET domain containing 2	*HYPB; HSPC069*	NP_054878	3p21.31	H3K36	16118227
SETD5	SET domain containing 5		BAB21848	3p25.3	n.a.	
SETD6	SET domain containing 6		NP_079136	16q21	n.a.	
SETD7	SET domain containing (lysine methyltransferase) 7	*SET7; SET7/9; SET9*	NP_085151	4q28	H3K4	11779497, 11850410
SETD8	SET domain containing (lysine methyltransferase) 8	*PR-Set7; SET07; SET8*	NP_065115	12q24.31	H4K20	12121615, 12086618, 12208845
SETDB1	SET domain, bifurcated 1	*ESET*	NP_036564	1q21	H3K9	11959841
SETDB2	SET domain, bifurcated 2		NP_114121	13q14	n.a.	
SETMAR	SET domain and mariner transposase fusion gene		NP_006506	3p26.2	H3K4, K36	16332963
SMYD1	SET and MYND domain containing 1	*BOP*	NP_938015	2p11.2	n.a.	
SMYD2	SET and MYND domain containing 2		NP_064582	1q41	H3K36	16805913
SMYD3	SET and MYND domain containing 3		NP_073580	1q44	H3K4	15235609
SMYD4	SET and MYND domain containing 4		AAH35077	17p13.3	n.a.	
SMYD5	SMYD family member 5		AAB38131	2p13.2	n.a.	
SUV39H1	suppressor of variegation 3-9 homolog 1 (Drosophila)		NP_003164	Xp11.23	H3K9	10949293
SUV39H2	suppressor of variegation 3-9 homolog 2 (Drosophila)		NP_078946	10p13	H3K9	11094092
SUV420H1	suppressor of variegation 4-20 homolog 1 (Drosophila)		NP_057112	11q13.2	H4K20	15145825
SUV420H2	suppressor of variegation 4-20 homolog 2 (Drosophila)		NP_116090	19q13.42	H4K20	15145825
WHSC1	Wolf-Hirschhorn syndrome candidate 1	*MMSET; NSD2*	NP_579877	4p16.3	n.a.	
WHSC1L1	Wolf-Hirschhorn syndrome candidate 1-like 1	*NSD3*	NP_075447	8p11.2	H3K4, K27	16682010
Drosophila melanogaster						
Ash1	absent, small, or homeotic discs 1		NP_524160	3L	H3K4, K9, K27	13679578, 12397363
Blimp-1	Blimp-1		NP_647982	3L	n.a.	
CG11160	CG11160		NP_727478	X	n.a.	
CG12119	CG12119		NP_572539	X	n.a.	
CG14122	CG14122		NP_648574	3L	n.a.	
CG14590	CG14590		NP_610202	2R	n.a.	
CG17086	CG17086		NP_609464	2L	n.a.	
CG1716	CG1716		NP_572888	X	n.a.	
CG18136	CG18136		NP_649084	3L	n.a.	
CG1868	CG1868		NP_724802	2R	n.a.	
CG2995	CG2995		NP_569834	X	H3K9, K27, H4?	16963494
CG30426	CG30426		NP_726483	2R	H3K9	17164421

Table S1. (Continued)

Gene Name	Description	Other Aliases	GenBank Accession Number	Chromosomal Location	HMT activity	Reference (PMID)
CG3353	CG3353		NP_650955	3R	n.a.	
CG40351	CG40351		EAL24598	n.a.	n.a.	
CG4565	CG4565		NP_650024	3R	n.a.	
CG8378	CG8378		NP_610730	2R	n.a.	
CG8503	CG8503		NP_610944	2R	n.a.	
CG9007	CG9007		NP_648681	3L	n.a.	
CG9640	CG9640		NP_611181	2R	n.a.	
CG9642	CG9642		NP_611182	2R	n.a.	
E(z)	Enhancer of zeste		NP_524021	3L	H3K27	12408864, 12408863
Mes-4	Mes-4		NP_733239	3R	n.a.	
*msta-A**	msta CG33548-PA		NP_001014717	X	n.a.	
*msta-B**	msta CG33548-PB		NP_001014718	X	n.a.	
pr-set7	pr-set7	*dSET8*	NP_731901	3R	H4K20	12121615, 12086618
Su(var)3-9	Suppressor of variegation 3-9		NP_524357	3R	H3K9	10949293
Suv4-20	Suv4-20		NP_569853	X	H4K20	15145825
trr	trithorax-related		NP_726773	X	H3K4	14603321
trx	trithorax		NP_599108	3R	H3K4	14730313
Caenorhabditis elegans						
blmp-1	Blimp1 (B Lymphocyte-induced Maturation Protein-1) homolog		NP_492723	I	n.a.	
C07A9.7	C07A9.7		NP_871669	III	n.a.	
C43E11.13	C43E11.13		NP_001021038	I	n.a.	
C47E8.8	C47E8.8		NP_506629	V	n.a.	
C49F5.2	C49F5.2		NP_510003	X	n.a.	
F02D10.7	F02D10.7		NP_510241	X	n.a.	
F15E6.1	F15E6.1		NP_500539	IV	n.a.	
F34D6.4	F34D6.4		NP_494334	II	n.a.	
K09F5.5	K09F5.5		NP_509306	X	n.a.	
K12H6.11	K12H6.11		NP_494371	II	n.a.	
lin-59	abnormal cell LINeage family member (lin-59)		NP_491206	I	n.a.	
mes-2	Maternal Effect Sterile		NP_496992	II	H3K27	15380065
mes-4	Maternal Effect Sterile		NP_506333	V	H3K36	16968818
met-2	histone METhyltransferase-like		NP_498848	III	n.a.	
R11E3.4	R11E3.4		NP_500642	IV	n.a.	
set-1	SET (trithorax/polycomb) domain containing		NP_001022796	III	n.a.	
set-2	SET (trithorax/polycomb) domain containing		NP_498039	III	n.a.	
T21B10.5	T21B10.5		NP_495902	II	n.a.	
tag-328	tag-328		NP_492529	I	n.a.	
tag-337	tag-337		NP_495272	II	n.a.	
tag-338	tag-338		NP_506569	V	n.a.	
tag-350	tag-350		NP_499819	III	n.a.	
W01C8.3	W01C8.3		NP_508956	X	n.a.	
W01C8.4	W01C8.4		NP_508957	X	n.a.	
Y24D9A.2	Y24D9A.2		NP_500555	IV	n.a.	
Y32F6A.1	Y32F6A.1		NP_505681	V	n.a.	
Y41D4B.12	Y41D4B.12		NP_741320	IV	n.a.	
Y43F11A.5	Y43F11A.5		NP_496579	II	n.a.	
Y51H4A.12	Y51H4A.12		NP_502971	IV	n.a.	
Y73B3B.2	Y73B3B.2		NP_508049	X	n.a.	
Schizosaccharomyces pombe						
clr4	histone H3 methyltransferase		NP_595186	II	H3K9	10949293
SPAC22E12.11c	hypothetical protein		NP_594837	I	n.a.	
SPAC29B12.02c	hypothetical protein	*Set2*	NP_594980	I	H3K36	16087749
SPAC3C7.09	hypothetical protein		NP_593610	I	n.a.	
SPBC16C6.01c	hypothetical protein		NP_001019057	II	n.a.	
SPBP8B7.07c	hypothetical protein		NP_596514	II	n.a.	
SPCC1223.04c	hypothetical protein		NP_588349	III	n.a.	
SPCC1739.05	hypothetical protein		NP_588413	III	n.a.	
SPCC297.04c	hypothetical protein		NP_588361	III	n.a.	
SPCC306.04c	hypothetical protein	*Set1*	NP_587812	III	H3K4	12193658, 12488447, 12589755
SPCC4B3.12	hypothetical protein	*Set9*	NP_588078	III	H4K20	15550243
Saccharomyces cerevisiae						
SET1	Histone methyltransferase, subunit of the COMPASS (Set1C) complex which methylates histone H3 on lysine 4; required in transcriptional silencing near telomeres and at the silent mating type loci; contains a SET domain		NP_011987	VIII	H3K4	11742990, 11751634, 11805083

Table S1. (Continued)

Gene Name	Description	Other Aliases	GenBank Accession Number	Chromosomal Location	HMT activity	Reference (PMID)
SET2	Histone methyltransferase with a role in transcriptional elongation, methylates a lysine residue of histone H3; associates with the C-terminal domain of Rpo21p; histone methylation activity is regulated by phosphorylation status of Rpo21p		NP_012367	X	H3K36	11839797
SET3	Defining member of the SET3 histone deacetylase complex which is a meiosis-specific repressor of sporulation genes; necessary for efficient transcription by RNAPII; one of two yeast proteins that contains both SET and PHD domains		NP_012954	XI	n.a.	
SET4	Protein of unknown function, contains a SET domain		NP_012430	X	n.a.	
SET5	Zinc-finger protein of unknown function, contains one canonical and two unusual fingers in unusual arrangements; deletion enhances replication of positive-strand RNA virus		NP_012077	VIII	n.a.	
SET6	Protein of unknown function; deletion heterozygote is sensitive to compounds that target ergosterol biosynthesis, may be involved in compound availability		NP_015160	XVI	n.a.	
SET7	Nuclear protein that contains a SET-domain	*RMS1*	NP_010543	IV	n.a.	

Note that we named the SET domain genes according to the current nomenclature in the Entrez Gene. Meanwhile, some other frequently used names of these genes were also listed as "Other Aliases".

*Drosophila proteins msta-A and msta-B are encoded by two alternative splicing isoforms of msta gene, and notably, they contain different SET domains.

n.a., not available.
