# Supplementary material for: Genome-Wide Survey and Developmental Expression Mapping of Zebrafish SET Domain-Containing Genes
Source: PLoS One. 2008 Jan 30;3(1):e1499. doi: 10.1371/journal.pone.0001499 (PMC2200798; doi:10.1371/journal.pone.0001499)
Supplement: Table S2 — Comparison of the expression patterns of zebrafish genes with their mouse counterparts revealed by mRNA in situ hybridization assays (0.12 MB PDF) [file pone.0001499.s008.pdf]

Table S2. Comparison of the expression patterns of zebrafish genes with their mouse countparts revealed by mRNA *in situ* hybridization assays.

Zebrafish Gene	mRNA *in situ* hybridization (whole-mount)	Mouse Gene	mRNA *in situ* hybridization (whole-mount)	mRNA *in situ* hybridization (section)
ezh1	Ubiquitous.	*Ezh1*	n.a.	Present in all tissues tested.
ezh2	Ubiquitous. 24-72 hpf: highly expressed in central nervous system, intermediate cell mass of mesoderm, and somites (see Figure S5 and text for details).	*Ezh2*	n.a.	E9.5: ubiquitous. E11.5: present in all tissues tested. E15.5: present in thymus primordium.
mll	Ubiquitous. 24 hpf: highly expressed in central nervous system and intermediate cell mass of mesoderm.	*Mll1*	E10.5: not specified.	E13.5: detected in neural and non-neural tissues; restricted in some cells (cerebral cortex, corpus striatum, spinal cord, etc.) of central nerous system.
mll3a	Ubiquitous. 24 hpf: highly expressed in intermediate cell mass of mesoderm.	*Mll3*	E3.5: inner cell mass. E10.5: not specified.	E13.5: not specified.
mll4a, mll4b	Ubiquitous.	*Wbp7*	E3.5: ubiquitous.	E16.0-adult: detected in all tissues tested.
nsd1a, nsd1b	Ubiquitous.	*Nsd1*	E10.5: not specified.	E13.5: not specified; detected in neural and non-neural tissues.
prdm1a	9 hpf: prechordal plate. 12 hpf: prechordal plate, somite, adaxial cells, intermediate mesoderm. 18 hpf: branchial arch, hatching gland, somite, tail epidermis. 24 hpf: branchial arch, fin buds, somite, cloaca, tail epidermis. 48-72 hpf: retina, pectoral fin, cloaca. 120 hpf: retina, pectoral fin.	*Prdm1*	E10.5: not specified.	E13.5: regionally restricted; absent in neural tissues.
prdm15	18 hpf: hatching gland, somite, muscle pioneer, neurons. 24 hpf: somite, muscle pioneer, neurons, and intermediate cell mass of mesoderm (see Figure 5 and 6 and text for detials).	*Prdm15*	E10.5: not specified.	E13.5: regionally restricted; present in thalamus, hypothalamus, midbrain.
prdm16	12 hpf: hindbrain. 18-24 hpf: forebrain, hindbrain, posterior pronephric duct. 48-72 hpf: olfactory bulb, hindbrain, pectoral fin bars (see Figure 6 and text for details).	*Prdm16*	E10.5: regionally restricted; present in telencephalon and hindbrain.	E13.5: present in cerebral cortex, corpus striatum, thalamus, hypothalamus, hindbrain, ear, telencephalon and olfactory lobe.
prdm3	12 hpf: forebrain, intermediate mesoderm. 18-24 hpf: forebrain, midbrain, hindbrain, posterior pronephric duct. 48 hpf: forebrain, midbrain, hindbrain, pectoral fin bars. 72 hpf: hindbrain, pectoral fins (see Figure 6 and text for details).	*Evi1*	E10.5: not specified.	E13.5: present in hypothalamus, hindbrain, spinal cord, telencephalon and olfactory lobe.
prdm4	24-48 hpf: muscle and somites (see Figure 5 and text for details)	*Prdm4*	E10.5: not specified.	E13.5: absent in neural tissues.
prdm5	Ubiquitous but weak.	*Prdm5*	E10.5: not specified.	E13.5: absent in neural tissues.
prdm6	Ubiquitous.	*Prdm6*	n.a.	E11.5-E18.5: present in all tissues tested.
prdm8a, prdm8b	12-18 hpf: spinal chord. 24-48 hpf: spinal chord, hindbrain (*prdm8a*). 48 hpf: olfactory placode, tegmentum, cerebellum and retina (*prdm8b*) (see Figure 6 and text for details).	*Prdm8*	n.a.	E18.0-adult: present in retinal tissues.
setd1ba, setd1bb	Ubiquitous.	*Setd1b*	n.a.	E13.5: present in all tissues tested.
setd6	Ubiquitous.	*Setd6*	n.a.	E14.0-adult: present in all tissues tested.
smyd1a, smyd1b	12 hpf: somites and muscle. 18-48 hpf: somites, muscle and heart. 72-120 hpf: somites and muscle (*smyd1b*). 18-120 hpf: somites and muscle (*smyd1a*) (see Figure 5 and text for details).	*Smyd1*	n.a.	E9.0: present in heart.
suv39h1a, suv39h1b	Ubiquitous.	*Suv39h1*	E7.5-9.5: ubiquitous.	n.a.
whsc1	Ubiquitous.	*Whsc1*	E10.5: ubiquitous.	n.a.

n.a., not available.
